# Transforming Growth Factor‐β‐Mediated Fibrotic Remodeling Drives Chronic Kidney Disease in Methylmalonic Aciduria and Propionic Aciduria—Identification of a New Therapeutic Target

**DOI:** 10.1002/jimd.70111

**Published:** 2025-10-25

**Authors:** Karina A. Zeyer, Stefan Tholen, Oliver Schilling, Leonie Gerling, Marina Morath, Stefan Kölker, Alexander Nyström, Ute Spiekerkoetter, Anke Schumann

**Affiliations:** ^1^ Department of Pediatrics, Adolescent Medicine and Neonatology Faculty of Medicine, Medical Center – University of Freiburg Freiburg Germany; ^2^ Institute for Surgical Pathology, Medical Center – University of Freiburg/Medical Faculty – University of Freiburg Freiburg Germany; ^3^ Proteomics Platform – Core Facility (ProtCF) Medical Center – University of Freiburg/Medical Faculty – University of Freiburg Freiburg Germany; ^4^ Center for Pediatric and Adolescent Medicine, Division of Pediatric Neurology and Metabolic Medicine Heidelberg University, Medical Faculty of Heidelberg, Heidelberg University Hospital Heidelberg Germany; ^5^ Department of Dermatology Faculty of Medicine, Medical Center – University of Freiburg Freiburg Germany

## Abstract

Propionic aciduria (PA‐uria) and methylmalonic aciduria (MMA‐uria) are caused by defects in propionate catabolism. While chronic kidney disease (CKD) is a well‐established complication in MMA‐uria, renal involvement in PA‐uria has only come into focus more recently, and the underlying mechanisms remain poorly understood. We investigated human renal epithelial cells from patients with PA‐uria, MMA‐uria, and healthy controls under metabolic stress, induced by methylmalonic acid, methylcitric acid, high‐protein, or isoleucine/valine‐enriched media. Proteomic profiling revealed significant enrichment of extracellular matrix (ECM)‐related pathways in PA‐uria cells. Both PA‐uria and MMA‐uria cells exhibited increased deposition of fibronectin and collagen fibers, which were further amplified under metabolic stress conditions. Transforming growth factor beta (TGF‐β) signaling was identified as a key pro‐fibrotic pathway. Pharmacological inhibition of the TGF‐β receptor signaling normalized fibronectin and collagen deposition in both PA‐uria and MMA‐uria cells. Treatment with losartan, an angiotensin II type 1 receptor blocker known to modulate TGF‐β signaling, also reversed the enhanced ECM deposition. This is the first study to mechanistically link ECM remodeling and TGF‐β signaling to CKD pathogenesis in both PA‐uria and MMA‐uria. Our findings highlight fibrotic remodeling as a shared pathogenic feature and suggest that losartan, a widely available and well‐tolerated drug, could be repurposed to mitigate renal fibrosis in these disorders.

**Abbreviations**
CKD: chronic kidney disease
ECM: extracellular matrix
EMT: epithelial–mesenchymal transition
GO: gene ontology
I/V: isoleucine/valine
MC: methylcitric acid
MCM: methylmalonyl‐CoA mutase
MMA: methylmalonic acid
MMA‐uria: methylmalonic aciduria
PA‐uria: propionic aciduria
PCC: propionyl‐CoA carboxylase
qRT‐PCR: quantitative real‐time polymerase chain reaction
ROS: reactive oxygen species
TCA: tricarboxylic acid
TGF‐β: transforming growth factor beta

## 1 Introduction

Organic acidurias are a group of rare inherited metabolic disorders characterized by defects in the catabolism of amino acids, fatty acids, or other metabolites, leading to the accumulation of organic acids in tissues and body fluids. These conditions often result in life‐threatening metabolic crises, multi‐organ complications, and progressive organ dysfunction. Among them, propionic aciduria (PA‐uria, OMIM #606054) and methylmalonic aciduria (MMA‐uria, OMIM #251000) are two closely related disorders affecting the mitochondrial propionate catabolic pathway with overlapping metabolic features and clinical manifestations, but differences in the underlying enzymatic defect and metabolic profile.

PA‐uria is a rare, autosomal‐recessive metabolic disorder caused by pathogenic variants in the *PCCA* or *PCCB* genes, which encode the mitochondrial enzyme propionyl‐CoA carboxylase (PCC). This biotin‐dependent enzyme catalyzes a key step in the anaplerotic propionate pathway, channeling succinyl‐CoA from various precursors to the tricarboxylic acid (TCA) cycle. PCC deficiency impairs the breakdown of branched‐chain amino acids (isoleucine, valine, threonine, and methionine), odd‐chain fatty acids, cholesterol side chains, and propionyl‐CoA derived from propionate‐producing gut bacteria. This leads to the accumulation of disease‐specific, potentially toxic metabolites, including propionylcarnitine, (3‐hydroxy) propionic acid, and 2‐methylcitric acid [1, 2].

Clinically, PA‐uria is characterized by acute, life‐threatening metabolic crises and hyperammonemia, particularly during catabolic stress such as the neonatal period or infections [1, 2]. Current treatment remains exclusively symptomatic, comprising dietary protein restriction, ammonia‐scavenging agents, intermittent antibiotic therapy to reduce propionate‐producing gut bacteria, stimulation of ureagenesis with carbamylglutamate, and extracorporeal detoxification during acute decompensation. Long‐term complications include neurological and cardiac impairments, and more recently recognized, chronic kidney disease (CKD) [3, 4, 5].

CKD is also a well‐established complication of MMA‐uria (OMIM #251000), a related inborn error of metabolism caused by deficiency of the enzyme methylmalonyl‐CoA mutase (MCM). This 5′‐desoxyadenosylcobalamin‐dependent enzyme resides two steps downstream of PCC in the propionate catabolic pathway. In contrast to PA‐uria, patients with MMA‐uria accumulate methylmalonic acid, in addition to the same toxic intermediates observed in PA‐uria. Notably, up to 60% of MMA‐uria patients develop CKD during infancy [6]. Markedly reduced levels of MCM activity and concomitantly high urinary levels of methylmalonic acid are considered a major risk factor for early onset and rapid progression of renal dysfunction in these individuals.

Both disorders are associated with marked alterations in mitochondrial energy metabolism and citric acid cycle activity in renal epithelial cells [7, 8]. However, the pathophysiological mechanisms underlying CKD in PA‐uria remain incompletely understood, especially since the disease‐specific metabolites made responsible for CKD in MMA‐uria, such as methylmalonic acid, are absent. Contributing factors in PA‐uria may include the toxicity of other accumulating metabolites, oxidative stress, and excess reactive oxygen species (ROS), chronic inflammation, fibroblast activation, mitochondrial dysfunction, and epithelial‐to‐mesenchymal transition (EMT) [9, 10].

In order to investigate the effects of disease‐specific metabolites on renal epithelial cells, we conducted proteomic profiling of renal epithelial cells derived from PA‐uria patients exposed to either methylcitric acid or methylmalonic acid and compared them to healthy control cells.

We can here show for the first time that extracellular matrix (ECM) is a key pathway differentially regulated between patient and control cells and that the identification of the transforming growth factor beta (TGF‐β) as a central pro‐fibrotic mediator identifies a new treatment target. Our findings highlight losartan as a promising candidate for drug repurposing as a disease‐modifying therapy for CKD in PA‐uria and MMA‐uria. Given its well‐established safety profile in fibrotic conditions, including pediatric populations, losartan represents a viable and readily translatable therapeutic option for patients with these rare metabolic disorders.

## 2 Results

**FIGURE 1:**
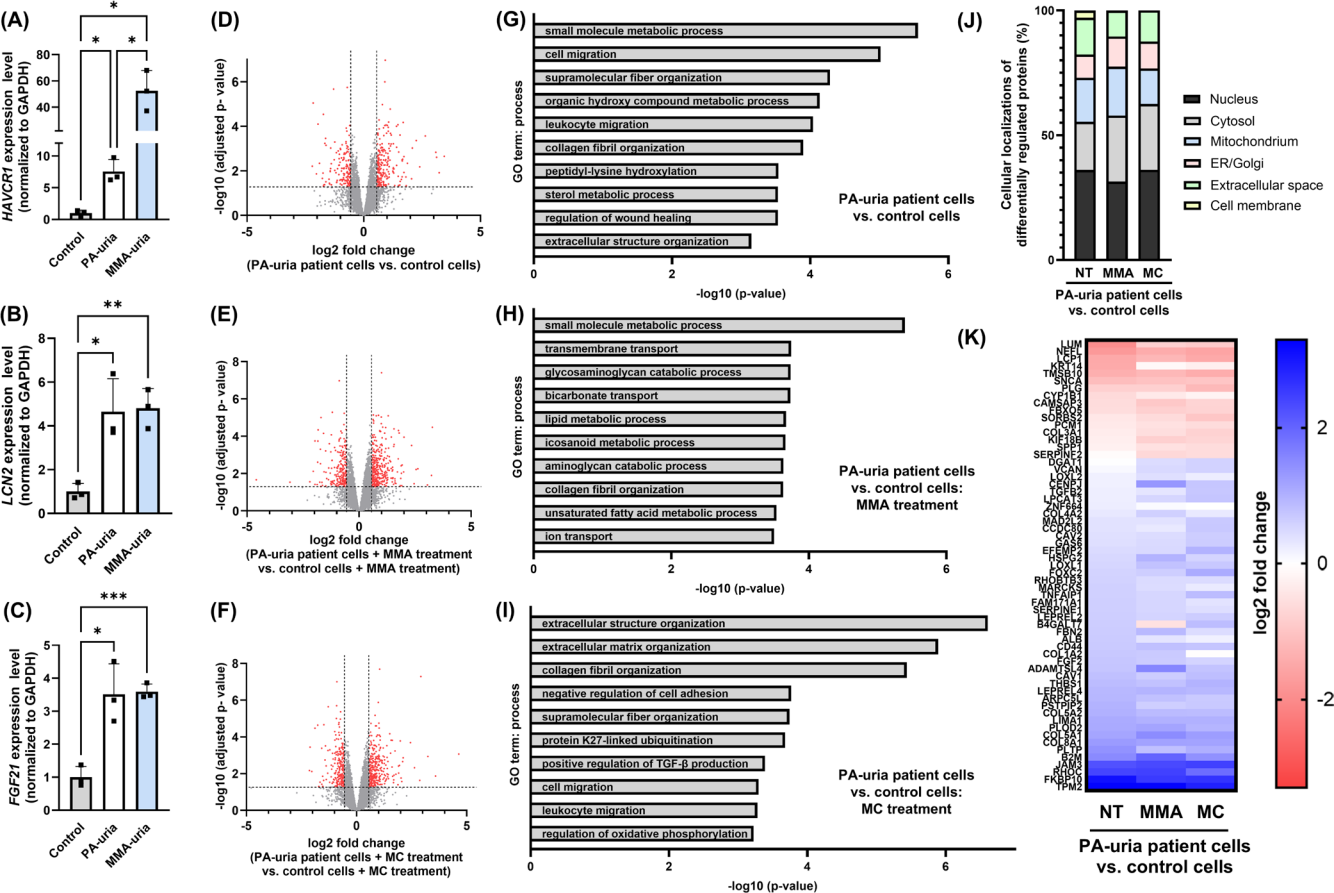
Proteomic analysis of PA‐uria renal epithelial cells. (A–C) Transcript levels of kidney injury molecule (KIM‐1, *HAVCR1*), lipocalin 2 (*LCN2*), and fibroblast growth factor 21 (*FGF21*) were assessed by real‐time quantitative PCR in renal epithelial cells from patients with PA‐uria or MMA‐uria and controls. *n* = 3 per group, data points represent biological replicates. (D–F) Volcano plot of mass spectrometry‐based proteomics data from PA‐uria patient renal epithelial cells compared to control cells. Cells were cultured with standard culture medium (D), or with the addition of methylmalonic acid (E) or methylcitric acid (F). *n* = 3 per group. In the pairwise comparison for indication of altered protein abundance, a combination of moderated *p* value (*p* value < 0.05) and log2 fold ≥ 0.58 and ≤ −0.58 was applied. (G–I) GO term analysis for the most significant and relevant GO terms for biological process for each condition. (J) The differentially regulated proteins were attributed to their subcellular localization. (H) Heatmap of differentially regulated proteins associated with GO terms of extracellular matrix.

We used an established cellular model of immortalized renal epithelial cells derived from the urine of PA‐uria and MMA‐uria patients [7, 8, 11, 12]. This model is suitable for studying CKD development, as shown by the upregulation of kidney injury markers such as kidney injury molecule‐1 (KIM‐1, *HAVCR1*), lipocalin‐2 (*LCN2*), and fibroblast growth factor 21 (*FGF21*) in patient cells (Figure 1A–C).

### 2.1 Proteomics Analysis Identifies a Central Role of the ECM in the Development of CKD in PA‐uria

To investigate the molecular and cellular mechanisms underlying the development of CKD in PA‐uria and the contribution of disease‐associated metabolites, we performed mass spectrometry‐based proteomic analysis on protein lysates from human renal epithelial cells derived from PA‐uria patients and healthy controls. Cells were cultured under three different conditions: (i) standard culture medium, (ii) exposure to methylmalonic acid, and (iii) exposure to methylcitric acid. While PA‐uria and MMA‐uria are metabolically related, methylmalonic acid accumulates only in MMA‐uria. Thus, using PA‐uria cells as a background allows the isolated study of the effects of methylmalonic acid in a system where it is not endogenously produced.

Across all conditions, several hundred proteins were differentially expressed between PA‐uria and control cells (Figure 1D–F). Gene ontology (GO) analysis revealed expected enrichment of terms related to metabolism (e.g., small molecule, sterol, lipid, and aminoglycan metabolism) (Figure 1G–I). Notably, GO terms associated with ECM organization, collagen fibril formation, wound healing, and cell migration were also enriched—processes that align with fibrotic remodeling observed in CKD. These ECM‐related GO terms were exclusively associated with upregulated proteins (Figure S1).

Interestingly, in cells exposed to methylcitric acid, proteins for mitochondrial pathways such as oxidative phosphorylation, electron transport chain, and NADH dehydrogenase complex assembly were downregulated. This indicates that methylcitric acid impairs mitochondrial energy production (Figure S1).

Subcellular localization analysis indicated many nuclear and cytosolic proteins, as expected, but also highlighted mitochondrial and extracellular proteins (Figure 1J). Treatment with methylmalonic acid or methylcitric acid only caused minor changes in the composition of subcellular localization.

A heatmap of ECM‐associated proteins showed consistent expression patterns across all conditions (Figure 1K), suggesting that exposure to methylcitric acid or methylmalonic acid does not further alter ECM‐related expression.

Our data identify the ECM as a previously undescribed pathway affected in PA‐uria patient cells and potentially involved in CKD development.

### 2.2 PA‐uria and MMA‐uria Renal Epithelial Cells Show Enhanced ECM Deposition

**FIGURE 2:**
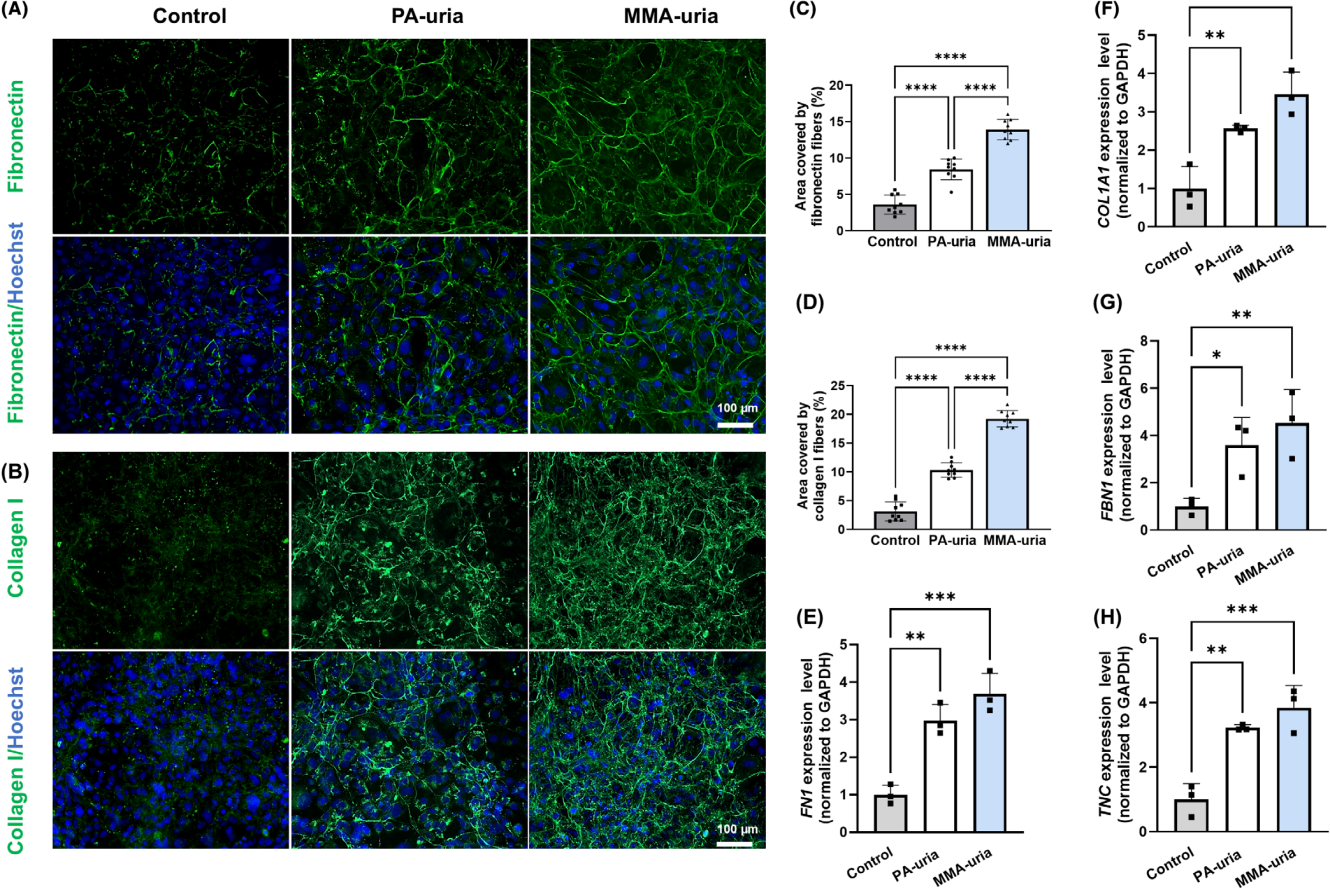
Fibronectin and collagen I deposition of PA‐uria and MMA‐uria renal epithelial cells. (A and B) Representative immunofluorescent staining for fibronectin (A) and collagen I (B) deposited by renal epithelial cells from PA‐uria or MMA‐uria patients and controls. Scale bar: 100 μm. *n* = 3. (C and D) Quantification of the area covered by the fiber network of fibronectin (C) or collagen I fibrils (D). Data points represent three technical replicates for each of three biological replicates. (E–H) Transcript levels of fibronectin (*FN1*, E), collagen I (*COL1A1*, F), fibrillin‐1 (*FBN1*, G), and tenascin C (*TNC*, H) were assessed by real‐time quantitative PCR in renal epithelial cells from patients with PA‐uria or MMA‐uria and controls. *n* = 3 per group, data points represent biological replicates.

To assess changes in the deposition of the ECM, we performed immunofluorescence staining for fibronectin and collagen. The collagen antibody was raised against collagen I, but reactivity against other collagens occurs. Fibronectin is typically deposited during injury and early fibrotic remodeling, while deposition of fibrillary collagen assemblies is a hallmark of advanced fibrosis [13, 14, 15]. As expected, control renal epithelial cells showed minimal pericellular ECM deposition without fibrillar structures (Figure 2A–D). In contrast, PA‐uria cells displayed markedly enhanced fibrillary fibronectin and collagen deposition.

For comparison, we included renal epithelial cells from MMA‐uria patients with early onset and severe kidney involvement [6]. These cells showed an even stronger fibrotic phenotype, with more extensive fibrillary fibronectin and collagen deposits than PA‐uria cells (Figure 2A–D). This increase in matrix deposition was accompanied by upregulated fibronectin (*FN1*) and collagen I alpha 1 chain (*COL1A1*) gene expression in both diseases, as confirmed by quantitative real‐time polymerase chain reaction (qRT‐PCR) (Figure 2E,F) and further indicative of wound healing and fibrosis‐related epithelial‐to‐mesenchymal phenotypic shift.

Interestingly, additional wound healing, fibrosis, and mesenchymal ECM components such as fibrillin‐1 (*FBN1*) and tenascin C (*TNC*) were also upregulated, indicating that excessive matrix deposition is a key feature of fibrosis in these cells (Figure 2G,H).

All lines retained a typical epithelial morphology, and cell numbers did not differ significantly between groups (Figure S2A,B). Additionally, expression of renal segment‐specific markers aquaporin 1 (AQP1) and 2 (AQP2), as well as epithelial (*CDH1*, e‐cadherin) and mesenchymal (*VIM*, vimentin) markers, remained unchanged (Figure S2C–F), supporting a preserved epithelial identity.

To determine whether EMT contributes to this accumulation of ECM, we analyzed the expression of EMT markers (*FOXC2*, *SNAI1*, *TWIST1*, and *ZEB1*) via qRT‐PCR. No significant differences were observed between patient and control cells (Figure S2G–J).

### 2.3 Metabolic Stress Through Protein Exposure Induces EMT in PA‐uria and MMA‐uria Renal Epithelial Cells

**FIGURE 3:**
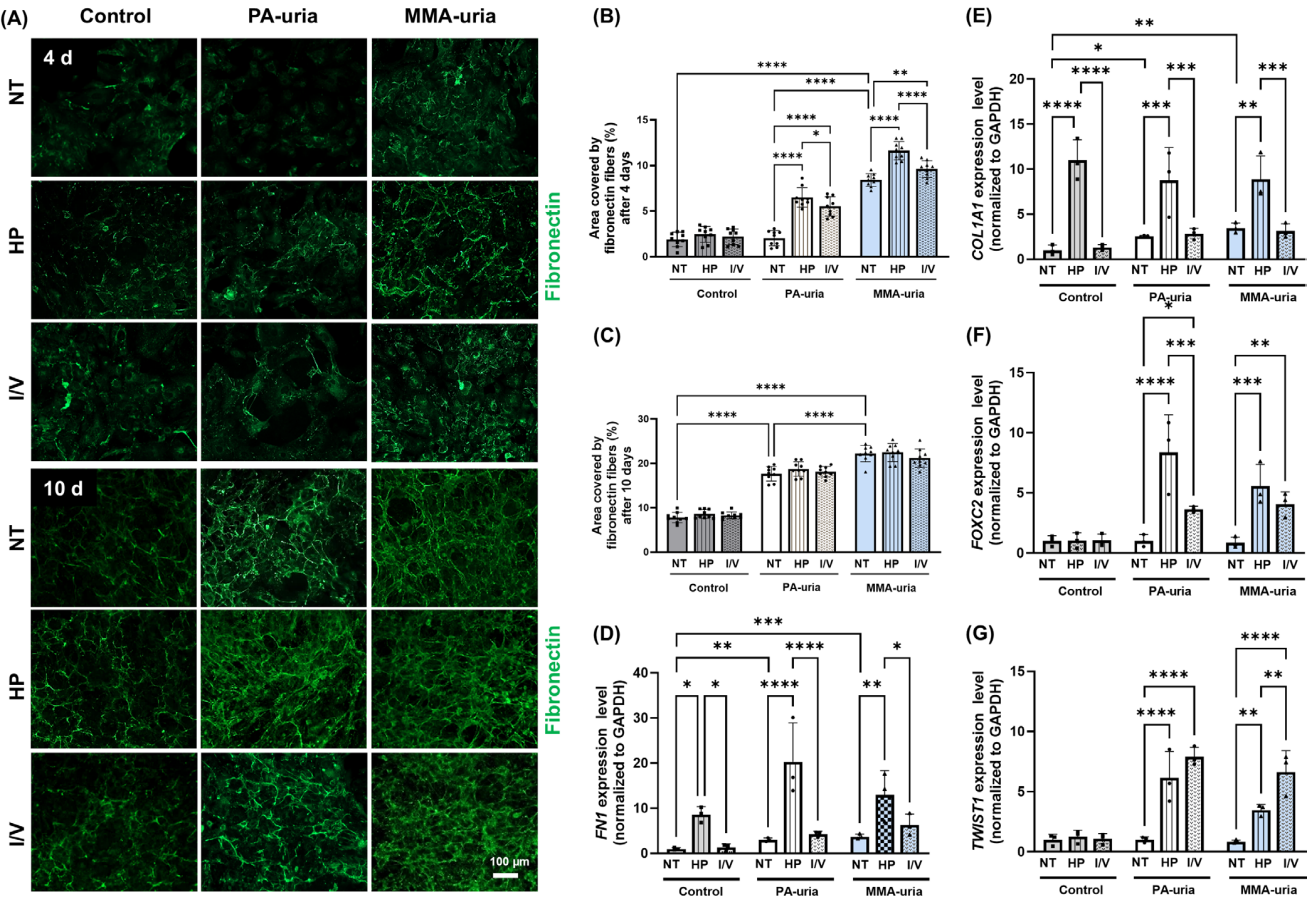
Impact of protein exposure on extracellular matrix deposition in PA‐uria and MMA‐uria renal epithelial cells. (A) Representative immunofluorescent staining for fibronectin deposited by renal epithelial cells from PA‐uria or MMA‐uria patients and controls after 4 days (upper panel) and 10 days (lower panel). Cells were exposed to low‐glucose medium enriched with high protein (HP) or with isoleucine/valine (I/V). As a control, cells were cultured under normal treatment (NT) conditions. Scale bar: 100 μm. *n* = 3. (B and C) Quantification of the area covered by the fibronectin fiber network of fibronectin after 4 days (B) or 10 days (C). Data points represent three technical replicates for each of three biological replicates. (D–G) Transcript levels of fibronectin (*FN1*, D), collagen I (*COL1A1*, E), forkhead box C2 (*FOXC2*, F), and twist 1 (*TWIST1*, G) were assessed by real‐time quantitative PCR in renal epithelial cells from patients with PA‐uria or MMA‐uria and controls. *n* = 3 per group, data points represent biological replicates.

To examine the impact of metabolic stress on ECM remodeling, renal epithelial cells were cultured in high‐protein (HP) medium or in medium enriched with isoleucine and valine (I/V), as previously described [7]. A low glucose medium was used to ensure that cells rely on amino acid metabolism and to mimic the clinical scenario during metabolic crisis in patients with hypoglycemia and catabolic protein load. After 4 days, fibronectin deposition was markedly elevated in MMA‐uria renal epithelial cells under HP conditions, while only a slight increase was observed under I/V conditions. PA‐uria renal epithelial cells showed a similar trend, though the overall fibronectin deposition was less pronounced compared to MMA‐uria cells. In contrast, control cells showed no fibronectin deposition under any of the tested conditions, including HP, I/V, or normal treatment (Figure 3A, upper panel; Figure 3B). Prolonged exposure (10 days) did not further enhance ECM accumulation as compared to untreated PA‐uria or MMA‐uria cells, suggesting a plateau in the fibrotic response (Figure 3A, lower panel; Figure 3C). The changes in fibronectin and collagen deposition were not reflected by changes in the number of cells, as evidenced by quantification of the nuclei (Figure S3).

qRT‐PCR analysis revealed increased transcription of fibronectin and collagen I in HP‐treated patient cells (Figure 3D,E). Interestingly, control cells also showed fibronectin and collagen I upregulation on the mRNA level under HP conditions, likely due to the higher fetal calf serum (FCS) content in the medium, which contains various growth factors known to induce matrix production. This was further supported by the upregulation of additional ECM components such as fibrillin‐1 and tenascin C (Figure S4A,B). In contrast, I/V treatment did not significantly induce fibronectin or collagen I transcription in control cells, indicating that enhanced deposition can occur independently of transcriptional changes and may reflect altered protein turnover or secretion.

In patient‐derived cells, both HP and I/V stress led to increased expression of EMT markers, suggesting that metabolic stress promotes a partial EMT, contributing to enhanced matrix deposition (Figures 3F,G and S4C,D). Functionally, this shift was supported by wound‐healing assays: PA‐uria and MMA‐uria cells demonstrated significantly increased migration under both stress conditions, consistent with a mesenchymal‐like phenotype (Figure S5).

In conclusion, these findings show that metabolic stress, particularly from protein overload, amplifies fibrotic remodeling in PA‐uria and MMA‐uria by enhancing matrix deposition and promoting a stress‐induced mesenchymal transition.

### 2.4 Methylmalonic Acid and Methylcitric Acid Contribute to Enhanced Deposition of Fibronectin and Collagen Fibers

**FIGURE 4:**
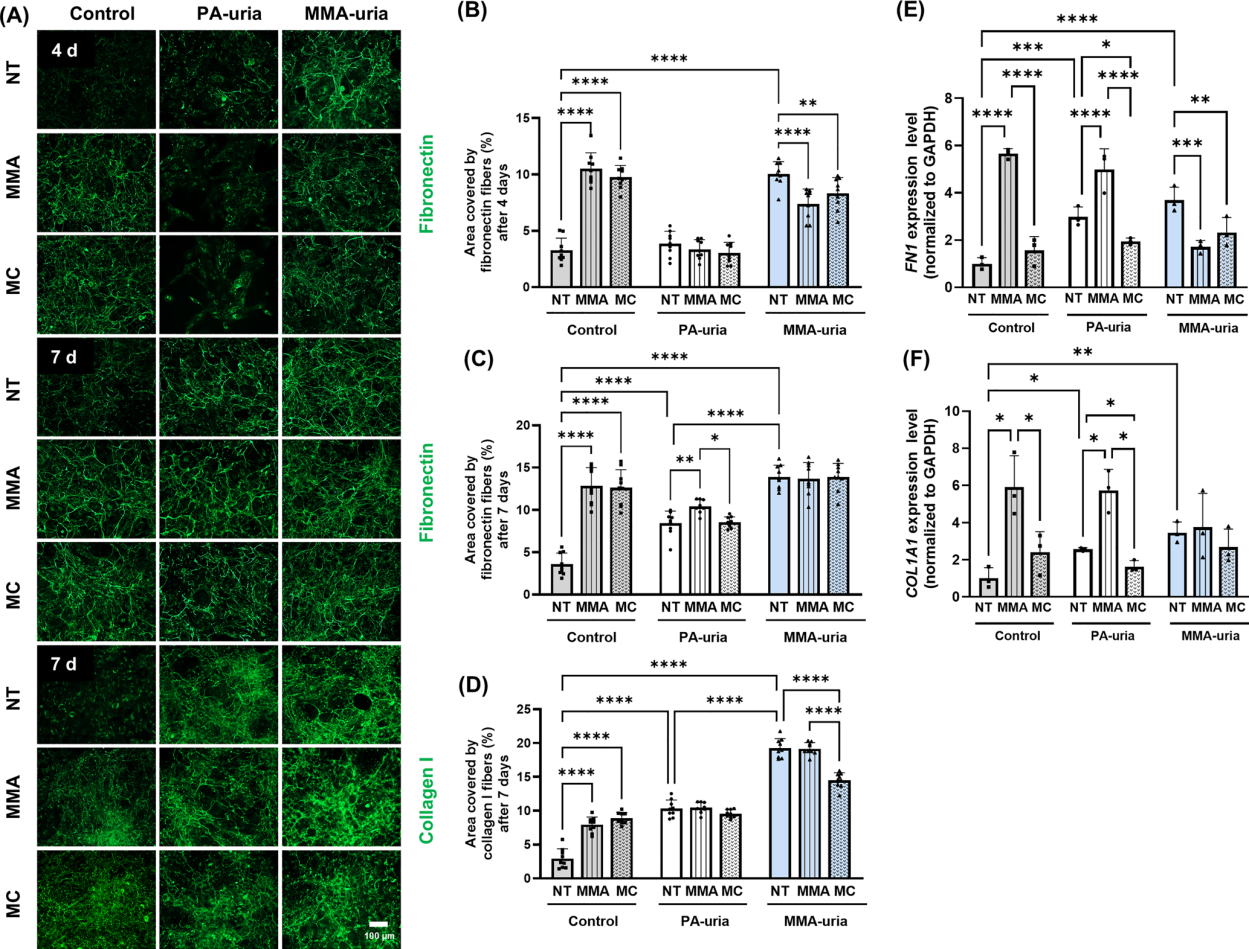
Impact of methylmalonic acid (MMA) and methylcitric acid (MC) on extracellular matrix deposition in PA‐uria and MMA‐uria renal epithelial cells. (A) Representative immunofluorescent staining for fibronectin or collagen I deposited by renal epithelial cells from PA‐uria or MMA‐uria patients and controls after 4 days (upper panel) and 7 days (fibronectin: Middle panel, collagen I: Lower panel). Cells were treated with either MMA or MC. As a control, cells were cultured under normal treatment (NT) conditions. Scale bar: 100 μm. *n* = 3 (B–D) Quantification of the area covered by the fibronectin fiber network of fibronectin after 4 days (B) or 7 days (C) or by collagen I fibers after 7 days (D). Data points represent three technical replicates for each of three biological replicates. (E and F) Transcript levels of fibronectin (*FN1*, E) and collagen I (*COL1A1*, F) were assessed by real‐time quantitative PCR in renal epithelial cells from patients with PA‐uria or MMA‐uria and controls. *n* = 3 per group, data points represent biological replicates.

Proteomic profiling revealed a significant enrichment of GO terms associated with ECM regulation in PA‐uria renal epithelial cells following exposure to methylcitric acid or methylmalonic acid. Correspondingly, exposure of healthy control renal epithelial cells to either metabolite led to robust fibrillary fibronectin and collagen deposits, suggesting a pro‐fibrotic potential of both metabolites. After 4 days, fibronectin deposition increased in control cells exposed to either metabolite but remained unchanged in PA‐uria cells (Figure 4A, upper panel; Figure 4B). In MMA‐uria cells, fibronectin deposition was slightly reduced upon treatment with methylcitric acid or methylmalonic acid. Notably, baseline fibronectin levels were already elevated in untreated MMA‐uria cells, suggesting that chronic exposure to endogenous methylmalonic acid and methylcitric acid may have already triggered fibronectin deposition. This implies that further external exposure does not amplify fibronectin deposition due to the persistent presence of these metabolites in the disease context.

After 7 days, fibronectin deposition remained elevated in control cells treated with methylmalonic acid or methylcitric acid as compared to untreated ones (Figure 4, middle panel; Figure 4C). In PA‐uria cells, exposure to methylmalonic acid—but not methylcitric acid—induced fibronectin accumulation, while MMA‐uria cells again showed no further increase. Similarly, collagen deposition was induced in control cells following exposure to either metabolite, but not in MMA‐uria or PA‐uria cells, except for a slight reduction with methylcitric acid in the latter (Figure 4, lower panel; Figure 4D). These findings point to a disease‐specific, metabolite‐saturated ECM response, where further stimulation does not enhance fibrotic outcomes.

Similar to the metabolic stress by protein exposure, the changes in ECM deposition were not caused by changes in the number of cells, as evidenced by quantification of the nuclei (Figure S6).

At the transcriptional level, *FN1* and *COL1A1* gene expression increased in control and PA‐uria cells following exposure to methylmalonic acid, consistent with protein deposition (Figure 4E,F). In contrast, exposure to methylcitric acid in PA‐uria cells reduced expression of both genes. In MMA‐uria cells, both metabolites reduced *FN1* expression, with no effect on *COL1A1*. Protein levels of fibronectin in PA‐uria cells varied among individuals, though a reduction was commonly observed following exposure to methylmalonic acid (Figure S7A,B). This suggests that fibronectin deposition might be regulated independently of total protein expression levels.

A similar pattern was observed for the expression of other ECM components, including fibrillin‐1 and tenascin‐C, which were upregulated in control cells following exposure to either metabolite (Figure S7C,D). Importantly, EMT markers remained unchanged under all conditions (Figure S7E–H), suggesting that ECM remodeling occurs partially independently of EMT activation.

In summary, exposure to methylmalonic acid and methylcitric acid promotes ECM remodeling in control cells, while responses in PA‐uria and MMA‐uria cells appear saturated or dysregulated due to chronic endogenous metabolite accumulation. These findings support a mechanistic role for these metabolites in driving fibrotic remodeling and CKD in PA‐uria and MMA‐uria.

### 2.5 Losartan Normalizes Fibronectin and Collagen Deposition in PA‐uria and MMA‐uria Renal Epithelial Cells

**FIGURE 5:**
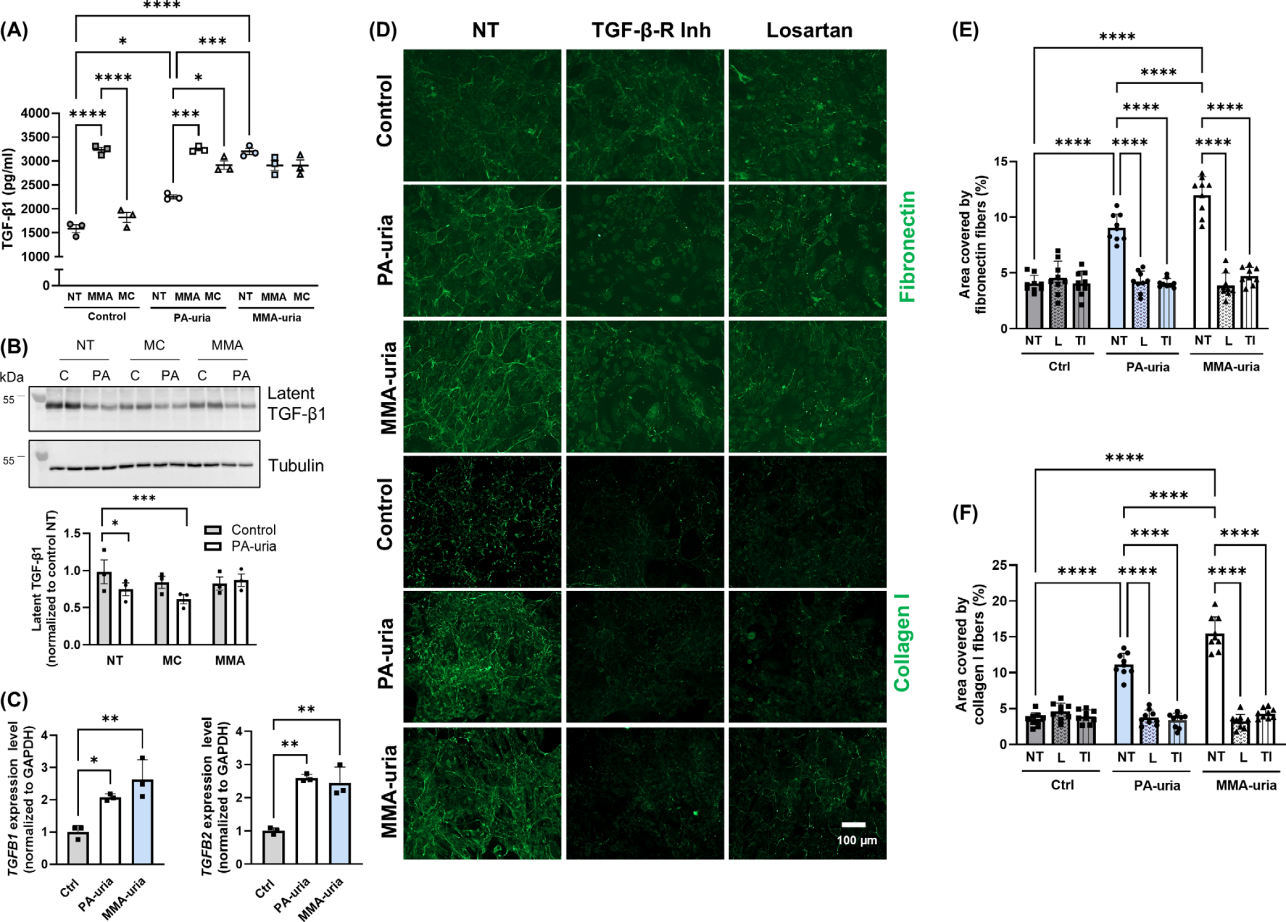
Losartan normalizes extracellular matrix deposition in PA‐uria and methylmalonic acid (MMA)‐uria renal epithelial cells. (A) TGF‐β ELISA of conditioned cell culture medium from PA‐uria and MMA‐uria patient and control cells after 7 days. Cells were treated with MMA or methylcitric acid (MC). As a control, cells were cultured under normal treatment (NT) conditions. *n* = 3, data points represent biological replicates. (B) Upper panel: Western blotting of TGF‐β1 in cell lysates from PA‐uria and control cells either exposed to MMA or MC or cultured under treatment conditions (NT). Lower panel: Densitometric quantification relative to untreated control cells. *n* = 3, data points represent biological replicates (C) Transcript levels of transforming growth factor beta 1 (*TGFB1*, left panel) and 2 (*TGFB2*, right panel) were assessed by real‐time quantitative PCR in renal epithelial cells from patients with PA‐uria or MMA‐uria and controls. *n* = 3, data points represent biological replicates. (D) Representative immunofluorescent staining for fibronectin or collagen I deposited by renal epithelial cells from PA‐uria or MMA‐uria patients and controls after 4 days (upper panel) and 7 days (fibronectin: Top panel, collagen I: Bottom panel). Cells were treated with either a dual TGF‐β receptor inhibitor (TI) or losartan (L). Scale bar: 100 μm. *n* = 3. (E and F) Quantification of the area covered by fibronectin fiber network (E) or collagen I fiber network (F) after 7 days. Cells were either cultured under regular conditions (NT) or treated with losartan (L) or a TGF‐β receptor inhibitor (TI). Data points represent three technical replicates for each of three biological replicates.

GO term analysis highlighted “regulation of TGF‐beta availability” as a significantly enriched term, with TGFB2 appearing in several ECM‐related categories. To investigate the role of TGF‐β signaling in ECM remodeling, we assessed its secretion levels in cell culture supernatants (Figure 5A). TGF‐β1 secretion was significantly elevated in both PA‐uria and MMA‐uria patient‐derived renal epithelial cells compared to control cells. This increase in secretion correlated with reduced latent TGF‐β1 protein levels, as demonstrated by Western blot analysis of cell lysates in PA‐uria cells (Figure 5B).

Interestingly, exposure to methylmalonic acid induced TGF‐β1 secretion not only in control cells but also in PA‐uria cells, which do not endogenously accumulate this metabolite (Figure 5A). Enhanced secretion was again accompanied by a decrease in intracellular latent TGF‐β1 protein levels (Figures 5B and S8). Moreover, qPCR analysis revealed upregulation of both *TGFB1* and *TGFB2* transcripts in baseline conditions, indicating transcriptional activation of the pathway (Figure 5C).

To assess the functional role of TGF‐β in the fibrotic remodeling in our cellular system, we inhibited its signaling capacity using a dual TGF‐β receptor inhibitor (Figure 5D–F). This intervention fully normalized fibronectin and collagen deposition to levels observed in control cells. While effective in vitro, complete inhibition of TGF‐β signaling is not possible in patients due to its critical homeostatic functions across multiple organ systems.

We therefore investigated losartan, an angiotensin II type 1 receptor blocker with known modulatory effects on TGF‐β signaling and established anti‐fibrotic properties (Figure 5D–F). Remarkably, treatment with losartan replicated the effects of direct TGF‐β receptor inhibition, significantly reducing ECM deposition. The changes in ECM deposition were not caused by changes in the number of cells, as evidenced by quantification of the nuclei (Figure S9).

Canonical TGF‐β signaling involves SMAD2/3 phosphorylation. Immunofluorescence staining revealed that both TGF‐β receptor inhibition and losartan treatment reduced levels of phosphorylated SMAD2/3 (Figure S10).

This suggests losartan as a potential therapeutic strategy for mitigating fibrosis in the development of CKD in PA‐uria and MMA‐uria.

## 3 Materials and Methods

### 3.1 Cell Culture

Human renal epithelial cells were isolated from spot urine samples obtained from healthy controls (*n* = 3) and patients with PA‐uria (*n* = 3) or MMA‐uria (*n* = 3). Cells were cultured in primary culture and subsequently immortalized after one passage by electroporation using an SV40 vector, as previously described [7, 8, 11, 12]. The cell lines were previously also referred to as human renal tubular (epithelial) cells [7, 8], kidney tubular cells [12], or human tubular epithelial cells (hTECs) [11]. The disease‐causing variants in the PA‐uria and MMA‐uria cell lines were described in previous studies [7, 8] and are listed in Table S1.

Cells were maintained in high‐glucose DMEM GlutaMAX medium (4.5 g/L glucose; Thermo Fisher Scientific) supplemented with 10% FCS (Biochrome), 100 U/mL penicillin–streptomycin (Thermo Fisher Scientific), and plasmocin (InvivoGen).

To minimize the risk of mycoplasma contamination, which can adversely affect renal epithelial cell physiology, the cells were cultured in the presence of plasmocin. All cell lines were tested routinely for mycoplasma contamination.

For metabolic stress experiments, cells were cultured in low‐glucose DMEM (1 g/L glucose) under two conditions for 7 days: (i) HP stress, consisting of 25% FCS; and (ii) I/V stress, consisting of 1000 μM isoleucine (Merck) and 3000 μM valine (Merck), 5% FCS. For the stress experiments with high protein or I/V stress, low‐glucose medium was used to ensure that cells rely on amino acid metabolism and to mimic the clinical scenario during metabolic crisis in patients with hypoglycemia and catabolic protein load.

For all other experiments, cells were cultured in standard high‐glucose medium to isolate and observe the specific effects of the added drug or metabolite.

For exposure experiments, methylmalonic acid (Merck) and methylcitric acid (Toronto Research Chemicals Inc.) were added to the culture medium at each media change, at concentrations of 1 mM. Pharmacological treatments included losartan (Merck) and the TGF‐β receptor inhibitor LY2109761 (MedChemExpress), each applied at a final concentration of 10 μM. Media were changed every 2–3 days. All experiments were conducted using cells between passages 6 and 15.

### 3.2 Proteomic Analysis

Cell lysis and preparation for mass spectrometry analysis were performed as described previously [16]. Peptides were analyzed with the Evosep One system (Evosep Biosystems, Odense, Denmark) coupled to a timsTOF fleX mass spectrometer (Bruker). A total of 500 ng of peptides was loaded onto Evotips C18 trap columns (Evosep Biosystems, Odense, Denmark) according to the manufacturer's protocol. Peptides were separated on an EV1137 performance column (15 cm × 150 μm, 1.5 μm, Evosep) using the standard implemented 30 SPD method with a gradient length of 44 min (buffer A: 0.1% v/v formic acid, dissolved in H_2_O; buffer B: 0.1% v/v formic acid, dissolved in acetonitrile). Over the time of the gradient, the concentration of acetonitrile gradually increased from 0% to 90% at a flow rate of 500 nL/min.

The timsTOF fleX mass spectrometer (Bruker, USA) was operated in the DIA‐PASEF mode. DIA MS/MS spectra were collected in an *m/z* range from 100 to 1700. Ion mobility resolution was set to 0.60–1.60 V·s/cm over a ramp time of 100 ms and an accumulation time of 100 ms. The cycle time was set at 1.8 s. The collision energy was programmed as a function of ion mobility, following a straight line from 20 eV for 1/K0 of 0.6 to 59 eV for 1/K0 of 1.6. The TIMS elution voltage was linearly calibrated to obtain 1/K0 ratios using three ions from the ESI‐L TuningMix (Agilent) (*m/z* 622, 922, and 1222).

Raw data were analyzed with DIA‐NN software (v.1.8.2 beta 22) [17]. A spectral library was predicted using a FASTA file containing the human protein sequences as of March 3, 2022 (human‐EBI‐reference database, https://www.ebi.ac.uk/). The false discovery rate (FDR) was set to 1%. The search was performed allowing one missed cleavage, and cysteine carbamidomethylation was enabled as a fixed modification. Match between runs was enabled. Quantification was performed using the label‐free quantification algorithm MaxLFQ, which calculates the protein quantities as ratios from all peptide intensities.

The data were further processed and analyzed for the statistical analysis using R (v4.3.0) within R Studio. Protein intensities were log2‐transformed and median‐normalized. Differential expression analysis was performed using limma [17]. *p* values were corrected by multiple testing using the Benjamini–Hochberg method, as applied by limma.

Mass spectrometry raw data have been deposited at the ProteomeXchange Consortium (http://proteomecentral.proteomexchange.org) under the accession number PXD066707. Furthermore, all mass spectrometry proteomics datasets used and/or analyzed during this study are available online at the MassIVE repository (http://massive.ucsd.edu/; dataset identifier: MSV000098667). This study did not generate new code for analysis.

In the pairwise comparison for indication of altered protein abundance, a combination of moderated *p* value (*p* value < 0.05) and log2 fold change ≥ 0.58 and ≤ −0.58, which relates to an alteration of 50%, was applied.

GO term analysis was conducted using the Gorilla web tool (Gene Ontology enRichment anaLysis and visuaLizAtion tool) to identify significantly enriched GO terms related to biological processes within a ranked list of proteins (Table S2) [18, 19].

### 3.3 Immunocytochemistry

Immunocytochemistry was performed at various time points to study the ECM. After washing once with PBS, cells were fixed with 70% methanol/30% acetone for 10 min (fibronectin, collagen I) or 4% PFA in PBS for 15 min (p‐SMAD2/3), followed by permeabilization with 0.5% Triton‐X (p‐SMAD2/3) in PBS for 50 min. Cells were then incubated with 3% BSA in PBS for 30 min to block nonspecific binding. Primary antibodies diluted in 3% BSA/PBS were applied for 1 h at room temperature: anti‐fibronectin (1:500, Abcam ab2413), anti‐collagen I (1:200, Acris R1038), and anti‐pSMAD2/3 (1:500, Abcam ab52903; Cell Signaling 138D4). After three PBS washes, Alexa Fluor 488‐conjugated goat anti‐rabbit secondary antibody (1:1000) was applied for 30 min. Nuclei were counterstained with Hoechst 3342 (1:2000) for 10 min, followed by mounting with fluorescence medium (Dako S3023).

### 3.4 RNA Extraction and Quantitative Real‐Time PCR

RNA was extracted from patient and control cells 7 days after seeding to study mRNA levels by quantitative real‐time PCR. Total RNA was extracted using the RNeasy Mini kit (QIAGEN) according to the manufacturer's instructions. A total of 200 000 cells were seeded per well of a six‐well plate for RNA extraction after 7 days.

The First Strand cDNA Synthesis Kit (Thermo Fisher Scientific, catalog number K1612) was used for the reverse transcription of mRNA, with a total of 2 μg of RNA used if RNA yields were sufficient, as described previously [20]. For real‐time qPCR, the CFX96 Real‐Time System (Bio‐Rad Laboratories) was used with SYBR green labeling, as described previously [20].

Primers are listed in Table S3.

### 3.5 Western Blotting

Western blotting was conducted to assess protein expression of fibronectin and TGF‐β1 in renal epithelial cells derived from PA‐uria patients and healthy controls. Cells were lysed in RIPA buffer containing protease inhibitors and then sonicated. Protein concentrations were measured using the BCA assay (Pierce, Thermo Fisher Scientific), and 20 μg of total protein per sample was resolved by SDS‐PAGE and transferred onto nitrocellulose membranes. Membranes were blocked in 5% nonfat dry milk in TBST and incubated overnight at 4°C with the following primary antibodies: anti‐fibronectin (1:1000, Abcam, ab2413), anti‐TGF‐β1 (1:1000, Abcam, ab315254), and anti‐γ‐tubulin (1:1000, Sigma, GTU‐88) as loading control. After washing, membranes were incubated for 2 h at 18°C with HRP‐conjugated secondary antibodies. Signal detection was performed using enhanced chemiluminescence (WBKLS0050, Millipore, Life Technologies), and band intensities were quantified using ImageJ software.

### 3.6 TGF‐β1 ELISA

Supernatant was collected from PA‐uria, MMA‐uria, and control renal epithelial cells after 7 days in culture. TGF‐β1 levels in the supernatant were quantified using the Human TGF‐β1 DuoSet ELISA Kit (R&D Systems, Minneapolis, MN, USA) following the manufacturer's protocol. Prior to measurement, all samples underwent acid activation to convert latent TGF‐β1 to its immunoreactive form. Briefly, 96‐well microplates were coated overnight at 4°C with a capture antibody specific for human TGF‐β1. After blocking nonspecific binding sites, activated samples, standards, and controls were added in duplicate and incubated at room temperature. A biotinylated detection antibody was then applied, followed by streptavidin‐horseradish peroxidase (HRP) conjugate. The signal was developed using a colorimetric substrate solution (TMB), and the reaction was stopped with sulfuric acid. Absorbance was measured at 450 nm with a correction at 540 nm using a microplate reader. TGF‐β1 concentrations were determined by interpolation from a standard curve generated with recombinant TGF‐β1 standards included in the kit.

### 3.7 Wound Healing Assay

PA‐uria, MMA‐uria, and control renal epithelial cells were seeded in six‐well plates and cultured until reaching confluency. A scratch was made across the cell monolayer using a sterile pipette tip. Images of the wound area were captured immediately after scratching (0 h), and then at 8 and 24 h. The width of the scratch was quantified at each time point to assess cell migration and wound closure. Experiments were performed in biological triplicate (*n* = 3).

### 3.8 Quantification of Fiber Network and Nuclei

Images were analyzed using ImageJ software. Fiber networks were quantified by measuring the percentage of area covered by fibers after thresholding to segment the fibrous structures. Nuclei were counted by applying a threshold to Hoechst‐stained images, followed by particle analysis to determine the number of nuclei per field. Multiple images per condition were analyzed to ensure accuracy and reproducibility.

### 3.9 Statistical Analysis

Data are presented as mean ± standard deviation. Two‐group comparisons were conducted using unpaired two‐tailed Student's *t*‐test. Before comparing more than two groups, data were first tested for variance homogeneity using an *F*‐test. For comparisons involving more than two groups, one‐way analysis of variance (ANOVA) was applied, followed by Tukey's post hoc test for multiple comparisons. In cases where variances among groups were significantly different, the Brown–Forsythe and Welch ANOVA test with Dunnett's test was used. Statistical analyses were performed using GraphPad Prism 9 software (https://www.graphpad.com). A *p* value of *p* < 0.05 was considered statistically significant. *p ≤ 0.05, **p ≤ 0.01, and ***p ≤ 0.001.

## 4 Discussion

**FIGURE 6:**
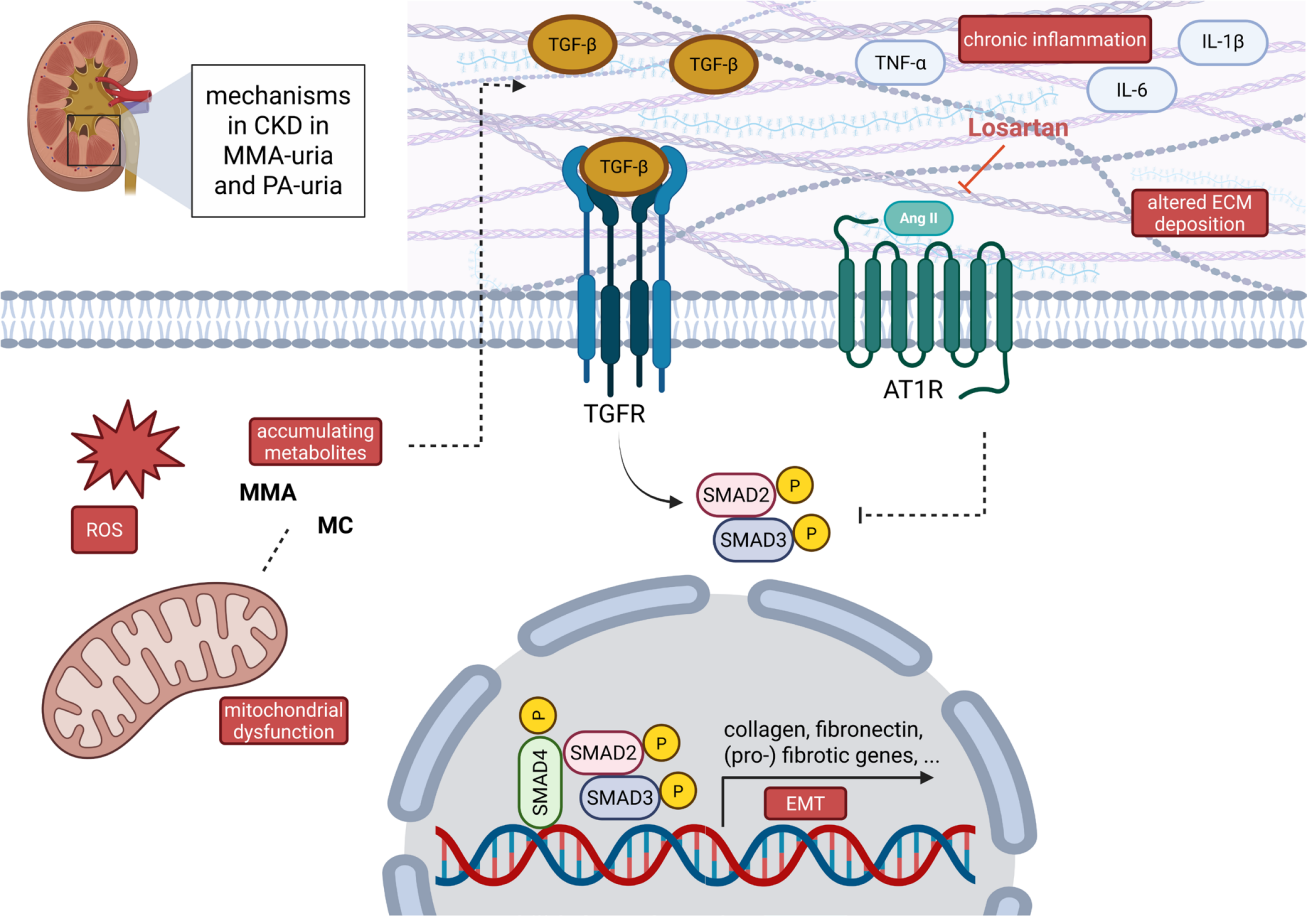
Losartan as a new therapeutic target in TGF‐β‐mediated fibrotic remodeling in chronic kidney disease in PA‐uria and MMA‐uria renal epithelial cells. The development of CKD in PA‐uria and MMA‐uria is driven by a complex interplay of factors, including the toxicity of accumulating metabolites, excess reactive oxygen species, chronic inflammation, mitochondrial dysfunction, epithelial‐to‐mesenchymal transition, and altered ECM deposition. In this study, we provide evidence that the TGF‐β‐mediated induction of (pro‐)fibrotic genes is, at least partially, dependent on SMAD2/3 signaling. Importantly, this pathway can be counteracted by losartan, an angiotensin II type 1 receptor blocker. Accumulating metabolites like MMA and MC are one factor that may contribute to increased TGF‐β levels through currently unidentified mechanisms. Losartan was able to normalize the pathological ECM deposition of fibronectin and collagen, suggesting that it may represent a novel preventive therapeutic strategy for managing CKD in patients with in MMA‐uria and PA‐uria. AT1R, angiotensin II type 1 receptor; CKD, chronic kidney disease; ECM, extracellular matrix; MC, methylcitic acid; MMA, methylmalonic acid; ROS, reactive oxygen species; TGFR, TGF‐β receptor. Created in BioRender. A. Schumann, 2025, https://BioRender.com/ss9lvvj.

CKD is an increasingly recognized long‐term complication in patients with PA‐uria, yet its pathogenesis has remained elusive. While CKD is a well‐characterized feature of MMA‐uria, with early onset and rapid progression, its delayed appearance and more variable course in PA‐uria have raised questions regarding shared and divergent mechanisms [3, 4, 5, 6]. In this study, we identify fibrotic ECM remodeling as a central and previously neglected feature of renal pathology in both PA‐uria and MMA‐uria. Using proteomic profiling and functional cell‐based assays, we demonstrate that disease‐specific metabolites—particularly methylcitric acid and methylmalonic acid—directly induce fibronectin and collagen I deposition in renal epithelial cells, and that this process is, at least in part, mediated by TGF‐β signaling (Figure 6).

These findings align with previous observations of renal fibrosis in other inborn errors of metabolism. Studies in MMA‐uria mouse models and patient tissue have documented tubular atrophy, interstitial fibrosis, and mitochondrial dysfunction in renal epithelial cells [12]. However, fibrosis has not yet been pathophysiologically explored in PA‐uria, and our data provide the first direct evidence that similar fibrogenic mechanisms are active in both disorders. The identification of ECM dysregulation and increased fibronectin/collagen deposition underscores the role of metabolite‐induced cellular stress as a common driver of fibrotic remodeling in PA‐uria and MMA‐uria.

Our data also contribute to the growing body of literature implicating mitochondrial dysfunction and oxidative stress in the renal pathology of metabolic disorders [12, 21, 22, 23].

Several studies have demonstrated that accumulating disease‐specific metabolites impair mitochondrial function through diverse mechanisms. In PA‐uria, propionyl‐CoA has been shown to inhibit the pyruvate dehydrogenase complex (PDHc), thereby preventing the conversion of pyruvate to acetyl‐CoA and limiting substrate flow into the TCA cycle [24]. Similarly, methylcitric acid interferes with multiple steps of the TCA cycle—namely citrate synthase, aconitase, and isocitrate dehydrogenase—resulting in broad suppression of mitochondrial energy metabolism, especially when combined with other toxic intermediates [25].

In MMA‐uria, methylmalonic acid has been shown to inhibit mitochondrial succinate transport, impairing both the TCA cycle and downstream electron transport chain activity [26]. This metabolic inhibition is compounded by evidence showing that methylmalonic acid and related intermediates promote oxidative stress, mitophagy defects, and epithelial damage [21].

Together, these findings underscore a multifaceted disruption of mitochondrial function in MMA‐uria and PA‐uria. This not only contributes to reduced ATP production and metabolic stress but also promotes pro‐fibrotic processes such as ROS generation, ECM deposition, and EMT, as observed in our in vitro model.

Although we did not directly assess mitochondrial function in this study, the fibrotic phenotype observed in patient‐derived cells was exacerbated under metabolic stress conditions—such as high protein or branched‐chain amino acid exposure—which are known to increase mitochondrial load and ROS production. Our study furthermore revealed 3.6‐fold elevated levels of *FGF21*, which has been identified as a highly predictive biomarker for disease severity and mitochondrial dysfunction in MMA‐uria patients [27]. It is therefore plausible that mitochondria‐derived ROS serve as upstream activators of TGF‐β signaling and ECM production, as described in other fibrotic kidney diseases [28]. This connection warrants further investigation, particularly in relation to antioxidant or mitochondria‐targeted therapies.

Notably, although EMT is widely reported as a contributor to renal fibrosis, we observed no significant EMT marker induction under basal conditions, despite robust fibronectin and collagen deposition. This suggests that ECM accumulation in PA‐uria and MMA‐uria may proceed through a non‐canonical or partial EMT program, in line with reports questioning full EMT in renal fibrosis [29]. However, under protein stress conditions, PA‐uria and MMA‐uria cells exhibited enhanced migration and EMT marker expression, supporting a stress‐induced shift toward a mesenchymal‐like phenotype. These findings highlight the context‐dependent nature of EMT in fibrotic processes and suggest that disease progression may be modulated by dietary or catabolic stressors.

The identification of TGF‐β as a central mediator of ECM remodeling in our model further supports its role as a key pathogenic driver for CKD. TGF‐β is well established as a pro‐fibrotic factor in both diabetic and non‐diabetic kidney disease [30, 31], and our data extend this paradigm to PA‐uria and MMA‐uria. Importantly, treatment with the angiotensin II type 1 receptor blocker losartan significantly reduced fibrillary fibronectin and collagen deposits in our cell models.

Interestingly, losartan was originally developed for hypertension but has since been recognized for its ability to modulate TGF‐β signaling, an important factor in fibrosis [32, 33, 34, 35]. Its potential to reduce fibrosis has been observed across several organ systems, such as the skin, heart, lung, liver, and kidneys, where it has been used successfully in treating fibrotic diseases [33, 36, 37, 38]. In addition to its broad clinical applications, losartan is also considered safe for use in pediatric populations. In pediatric cohorts, losartan has been tested in conditions such as Marfan syndrome, where it helps reduce aortic root dilation [39], and in recessive dystrophic epidermolysis bullosa, where it has shown promising effects in reducing fibrotic tissue formation [40]. Its safety in pediatric patients, including those with long‐term use, further strengthens its potential as a repurposed therapy for fibrotic diseases in young patients.

In our in vitro cellular model, losartan has been successfully used to prevent or attenuate the progression of fibrotic disease, rather than to treat advanced fibrosis. A similar effect might be expected in PA‐uria and MMA‐uria patients, where losartan may offer preventive benefits but may no longer be effective once advanced CKD is established.

Losartan may exert its anti‐fibrotic effects through downstream inhibition of SMAD2/3 activation. We observed trends toward reduced SMAD2/3 phosphorylation in PA‐uria and MMA‐uria cells treated with losartan. These findings align with the canonical model of TGF‐β‐driven fibrosis, wherein SMAD2/3 translocate to the nucleus to induce transcription of ECM‐related genes. Notably, TGF‐β can also activate non‐canonical signaling cascades, including p38 MAPK, JNK, and ERK pathways [41]; the latter has been implicated in fibrotic processes and may also be modulated by ARB treatment. While these results are promising, the mechanistic role of SMAD2/3 and the potential involvement of p‐ERK signaling remain speculative and are not the primary focus of this study, and will require further investigations.

Limitations of our study include the use of in vitro models that do not fully recapitulate the in vivo renal microenvironment, including interactions with immune cells, fibroblasts, and vascular components. Additionally, we focused on renal epithelial cells, though other cell types may also contribute to fibrosis and CKD progression.

In conclusion, our findings establish fibrotic ECM remodeling as a key pathophysiological mechanism in the development of CKD in both PA‐uria and MMA‐uria. We identify TGF‐β as a central mediator of this process and highlight losartan as a promising preventive therapeutic candidate. These results offer a new perspective on renal disease in PA‐uria and MMA‐uria and provide a compelling rationale for targeted anti‐fibrotic therapies in these patients.

## Author Contributions

**Karina A. Zeyer:** conceptualization, formal analysis, funding acquisition, investigation, writing – original draft, writing – review and editing. **Stefan Tholen:** formal analysis, investigation, writing – review and editing. **Oliver Schilling:** formal analysis, writing – review and editing. **Leonie Gerling:** formal analysis, investigation, writing – review and editing. **Marina Morath:** resources, writing – review and editing. **Stefan Kölker:** resources, writing – review and editing. **Alexander Nyström:** resources, writing – review and editing. **Ute Spiekerkoetter:** funding acquisition, supervision, writing – review and editing. **Anke Schumann:** conceptualization, formal analysis, funding acquisition, supervision, writing – original draft, writing – review and editing.

## Ethics Statement

The study was conducted in accordance with relevant ethical guidelines and approved by the appropriate institutional review boards of the Universities of Freiburg (Ethical vote: 23‐1467‐S1) and Heidelberg (Ethical vote for cell collection: S‐074/2008).

## Consent

All participants or their legal guardians provided written informed consent.

## Conflicts of Interest

The authors declare no conflicts of interest.

## Supporting information


**Figure S1:** (A–F) The GO term analysis for the most significant and relevant GO terms for biological process for each condition: standard culture medium (A and D), methylmalonic acid (MMA) treatment (B and E), and methylcitric acid (MC) treatment (C and F) is shown separated for up‐ (shown in green; A, C, and E) and downregulated (shown in red; B, D, and F) GO terms.

**Figure S2:** (A) Light microscopic images from PA‐uria, MMA‐uria, and control renal epithelial cells. Scale bar: 100 μm. (B) Quantification of the number of nuclei per picture of the immunofluorescent staining in Figure 2, as shown by Hoechst staining. Data points represent three technical replicates for each of three biological replicates. (C–J) Transcript levels of aquaporin 1 (*AQP1*, C), aquaporin 2 (*AQP2*, D), vimentin (*VIM*, E), e‐cadherin (*CDH1*, F), forkhead box C2 (*FOXC2*, G), snail (*SNAI1*, H), twist (*TWIST1*, I), and zinc‐finger‐enhanced binding protein 1 (*ZEB1*, J) were assessed by real‐time quantitative PCR renal epithelial cells from patients with PA‐uria or MMA‐uria and controls. *n* = 3 per group, data points represent biological replicates.

**Figure S3:** (A) The immunofluorescent images from Figure 3 are shown along with merged images of Hoechst staining to account for phenotypic changes through different cell numbers. (B and C) Quantification of the number of nuclei per picture of the immunofluorescent staining in Figure 3, as shown by Hoechst staining after 4 days (B) and 10 days (C). Data points represent three technical replicates for each of three biological replicates.

**Figure S4:** (A–D) Transcript levels of fibrillin‐1 (*FBN1*, A), tenascin C (*TNC*, B), snail (*SNAI1*, C), and zinc‐finger‐enhanced binding protein 1 (*ZEB1*, D) were assessed by real‐time quantitative PCR in renal epithelial cells from patients with PA‐uria or MMA‐uria and controls. *n* = 3 per group, data points represent biological replicates.

**Figure S5:** (A) Representative light microscopic images of the wound healing/cell migration assay are shown at the time of scratching (upper panel), after 8 h (middle panel), and after 24 h (lower panel). Red lines indicate the border between the cell‐covered area and the cell‐free (wound) area. Scale bar: 100 μm. (B–D) Quantification of the area devoid of cells at the time of wounding (B), after 8 h (C), and after 24 h (D). Data points represent three technical replicates for each of three biological replicates.

**Figure S6:** (A) The immunofluorescent images from Figure 4 are shown along with merged images of Hoechst staining to account for phenotypic changes through different cell numbers. (B–D) Quantification of the number of nuclei per picture of the immunofluorescent staining in Figure 4, as shown by Hoechst staining after 4 days (B) and 7 days (C) for the fibronectin staining and after 7 days (D) for the collagen I staining. Data points represent three technical replicates for each of three biological replicates.

**Figure S7:** (A) Western blotting of fibronectin in cell lysates from PA‐uria and control cells, either untreated (NT) or exposed to methylmalonic acid (MMA) or methylcitric acid (MC). Due to differences between patient cells, blots for each patient are shown. (B) Densitometric quantification of the Western blot in (A) relative to untreated control cells. *n* = 3. Data points represent three technical replicates for each of three biological replicates in two independent experiments. (C–H) Transcript levels of fibrillin‐1 (*FBN1*, C), tenascin C (*TNC*, D), forkhead box C2 (*FOXC2*, E), snail (*SNAI1*, F), and twist 1 (*TWIST1*, G) and T zinc‐finger‐enhanced binding protein 1 (*ZEB1*, H) were assessed by real‐time quantitative PCR renal epithelial cells from patients with PA‐uria or MMA‐uria and controls. Cells were either cultured under regular conditions (NT) or treated with MMA or MC. *n* = 3 per group, data points represent biological replicates.

**Figure S8:** Western blotting of TGF‐β1 in cell lysates from PA‐uria and control cells, either exposed to methylmalonic acid (MMA) or methylcitric acid (MC) or cultured under treatment conditions (NT). Blots from all three different patient PA‐uria cell lines and controls used are shown.

**Figure S9:** (A) The immunofluorescent images from Figure 5 are shown along with merged images of Hoechst staining to account for phenotypic changes through different cell numbers. (B and C) Quantification of the number of nuclei per picture of the immunofluorescent staining in Figure 4, as shown by Hoechst staining after 7 days for both the fibronectin (B) and collagen I (C) staining. Data points represent three technical replicates for each of three biological replicates.

**Figure S10:** Representative immunofluorescent staining for pSMAD2/3 in renal epithelial cells from PA‐uria or MMA‐uria patients and controls after 7 days. Cells were either cultured under regular conditions (NT) or treated with losartan (L) or a TGF‐β receptor inhibitor (TI). Scale bar: 50 μm. *n* = 3.

**Table S1:** The disease‐causing variants on the gene and protein level are shown for the MMA‐uria and PA‐uria cell lines used.

**Table S2:** Gene ontology (GO) term analysis was conducted using the Gorilla web tool (Gene Ontology enRichment anaLysis and visuaLizAtion tool) to identify significantly enriched GO terms related to biological processes within a ranked list of proteins. The selected GO terms used in Figures 1 and S1 are highlighted in red. GO terms were selected based on their biological relevance in the cellular system used in this study. If several closely related GO terms were identified, only one was chosen. Different spreadsheets show GO terms identified with either all differentially regulated proteins, or with only up‐ or downregulated proteins. (A–C) PA‐uria patient cells vs. control cells: GO terms for all differentially regulated proteins (A), upregulated (B), or downregulated proteins (C). (D–F) PA‐uria patient cells vs. control cells, MC treatment: GO terms for all differentially regulated proteins (D), upregulated (E), or downregulated proteins (F). (G–I) PA‐uria patient cells vs. control cells, MMA treatment: GO terms for all differentially regulated proteins (G), upregulated (H), or downregulated proteins (I).

**Table S3:** Sequences of forward and reverse primers used for real‐time quantitative PCR are listed for each gene.

## Data Availability

Mass spectrometry raw data have been deposited at the ProteomeXchange Consortium (http://proteomecentral.proteomexchange.org) under the accession number PXD066707. Furthermore, all mass spectrometry proteomics datasets used and/or analyzed during this study are available online at the MassIVE repository (http://massive.ucsd.edu/; dataset identifier: MSV000098667). This study did not generate new code for analysis.
