# Supplementary figures and images for: Transforming Growth Factor‐β‐Mediated Fibrotic Remodeling Drives Chronic Kidney Disease in Methylmalonic Aciduria and Propionic Aciduria—Identification of a New Therapeutic Target
Source: J Inherit Metab Dis. 2025 Oct 25;48(6):e70111. doi: 10.1002/jimd.70111 (PMC12553402; doi:10.1002/jimd.70111)

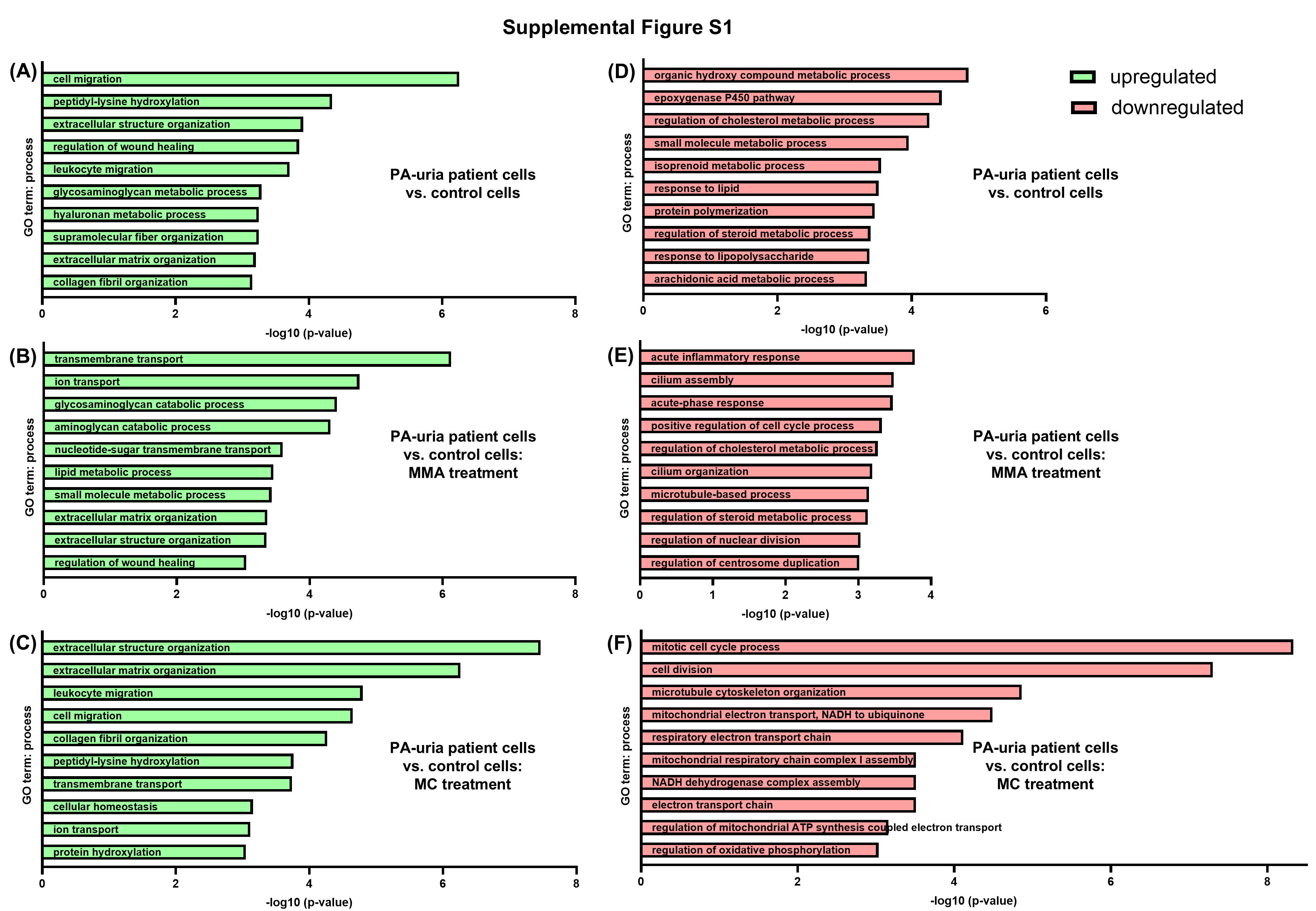

Supplement: Supplementary file 1 — Figure S1: (A–F) The GO term analysis for the most significant and relevant GO terms for biological process for each condition: standard culture medium (A and D), methylmalonic acid (MMA) treatment (B and E), and methylcitric acid (MC) treatment (C and F) is shown separated for up‐ (shown in green; A, C, and E) and downregulated (shown in red; B, D, and F) GO terms. [file JIMD-48-0-s012.png]

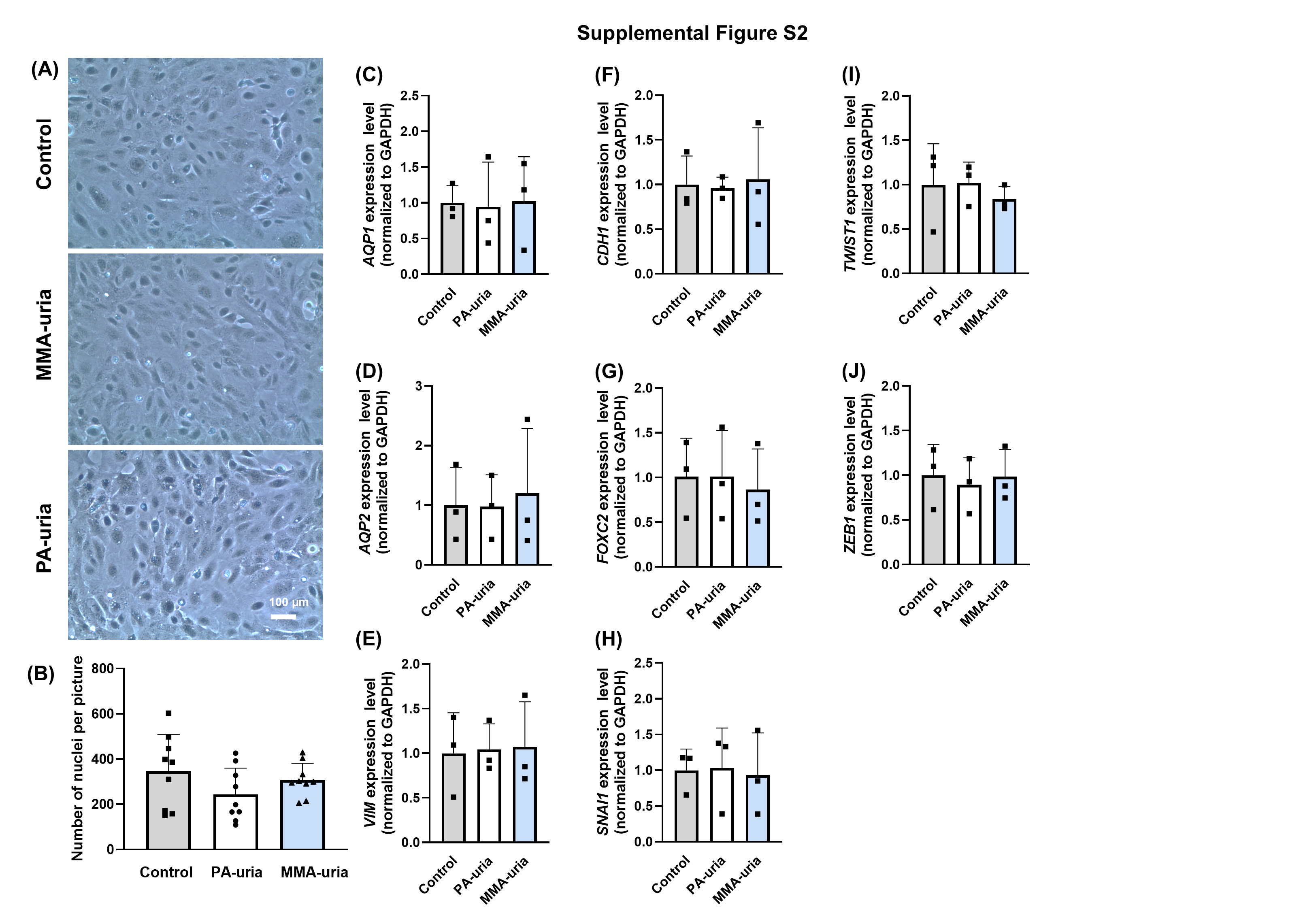

Supplement: Supplementary file 2 — Figure S2: (A) Light microscopic images from PA‐uria, MMA‐uria, and control renal epithelial cells. Scale bar: 100 μm. (B) Quantification of the number of nuclei per picture of the immunofluorescent staining in Figure 2, as shown by Hoechst staining. Data points represent three technical replicates for each of three biological replicates. (C–J) Transcript levels of aquaporin 1 (AQP1, C), aquaporin 2 (AQP2, D), vimentin (VIM, E), e‐cadherin (CDH1, F), forkhead box C2 (FOXC2, G), snail (SNAI1, H), twist (TWIST1, I), and zinc‐finger‐enhanced binding protein 1 (ZEB1, J) were assessed by real‐time quantitative PCR renal epithelial cells from patients with PA‐uria or MMA‐uria and controls. n = 3 per group, data points represent biological replicates. [file JIMD-48-0-s001.png]

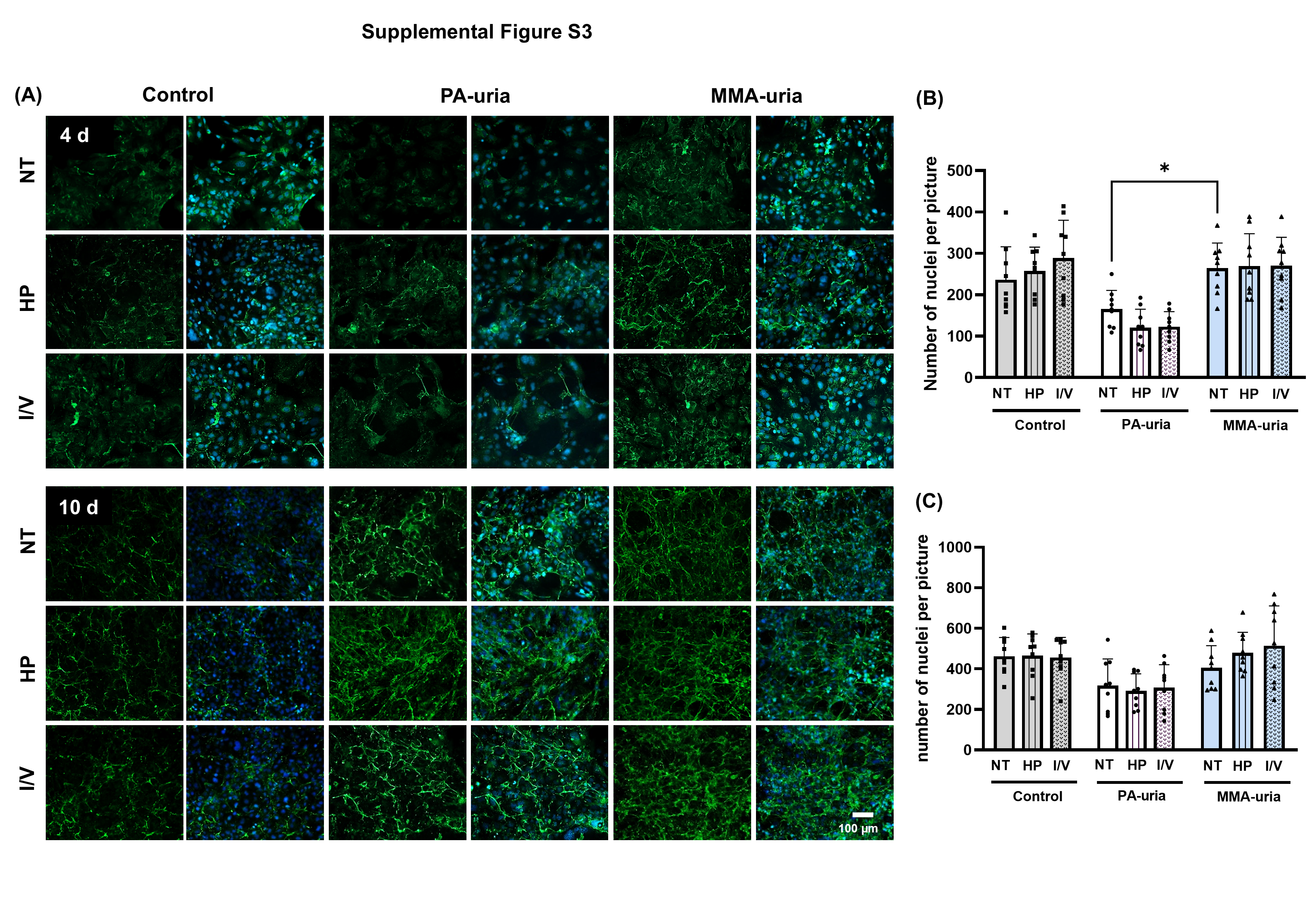

Supplement: Supplementary file 3 — Figure S3: (A) The immunofluorescent images from Figure 3 are shown along with merged images of Hoechst staining to account for phenotypic changes through different cell numbers. (B and C) Quantification of the number of nuclei per picture of the immunofluorescent staining in Figure 3, as shown by Hoechst staining after 4 days (B) and 10 days (C). Data points represent three technical replicates for each of three biological replicates. [file JIMD-48-0-s013.png]

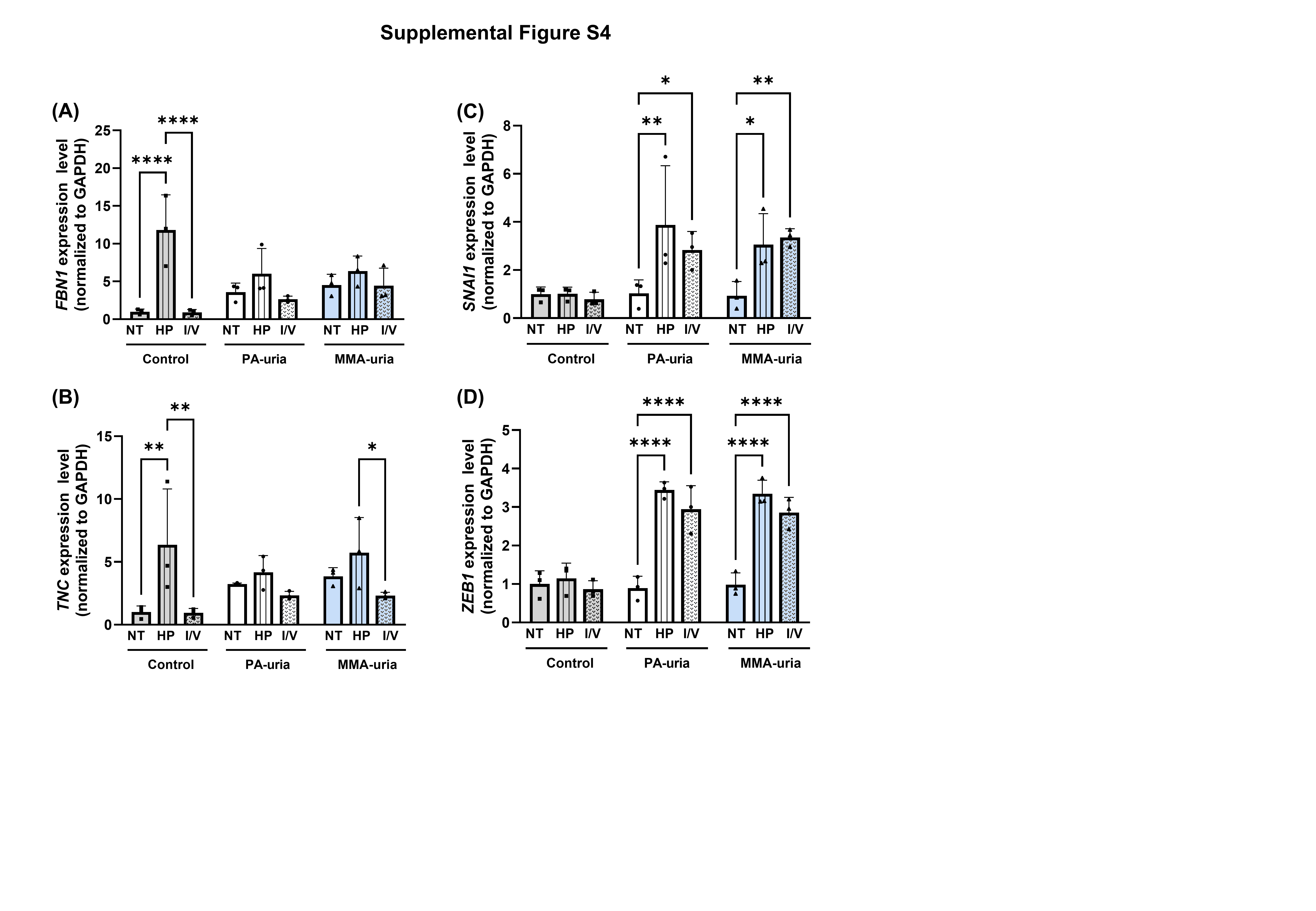

Supplement: Supplementary file 4 — Figure S4: (A–D) Transcript levels of fibrillin‐1 (FBN1, A), tenascin C (TNC, B), snail (SNAI1, C), and zinc‐finger‐enhanced binding protein 1 (ZEB1, D) were assessed by real‐time quantitative PCR in renal epithelial cells from patients with PA‐uria or MMA‐uria and controls. n = 3 per group, data points represent biological replicates. [file JIMD-48-0-s002.png]

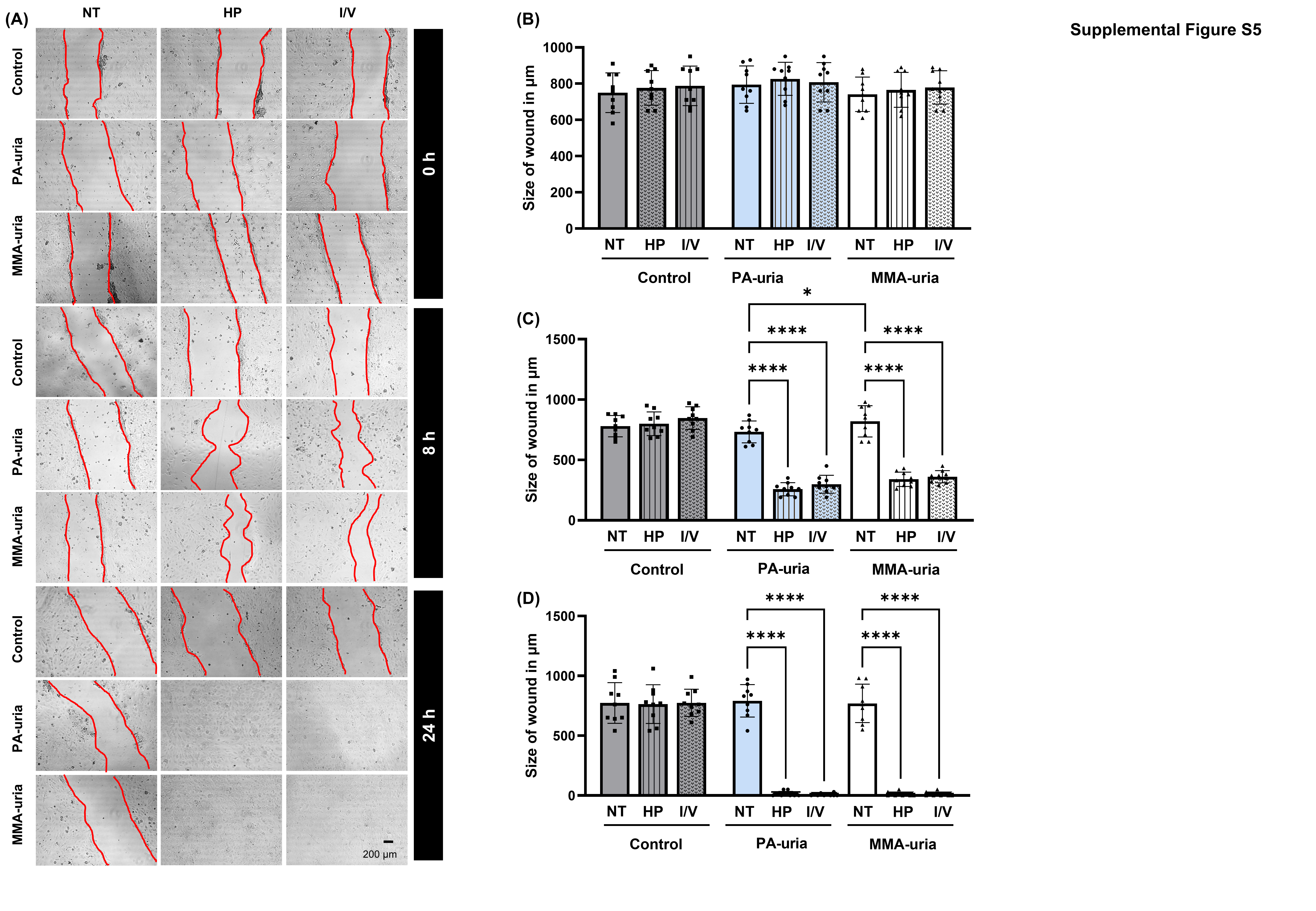

Supplement: Supplementary file 5 — Figure S5: (A) Representative light microscopic images of the wound healing/cell migration assay are shown at the time of scratching (upper panel), after 8 h (middle panel), and after 24 h (lower panel). Red lines indicate the border between the cell‐covered area and the cell‐free (wound) area. Scale bar: 100 μm. (B–D) Quantification of the area devoid of cells at the time of wounding (B), after 8 h (C), and after 24 h (D). Data points represent three technical replicates for each of three biological replicates. [file JIMD-48-0-s011.png]

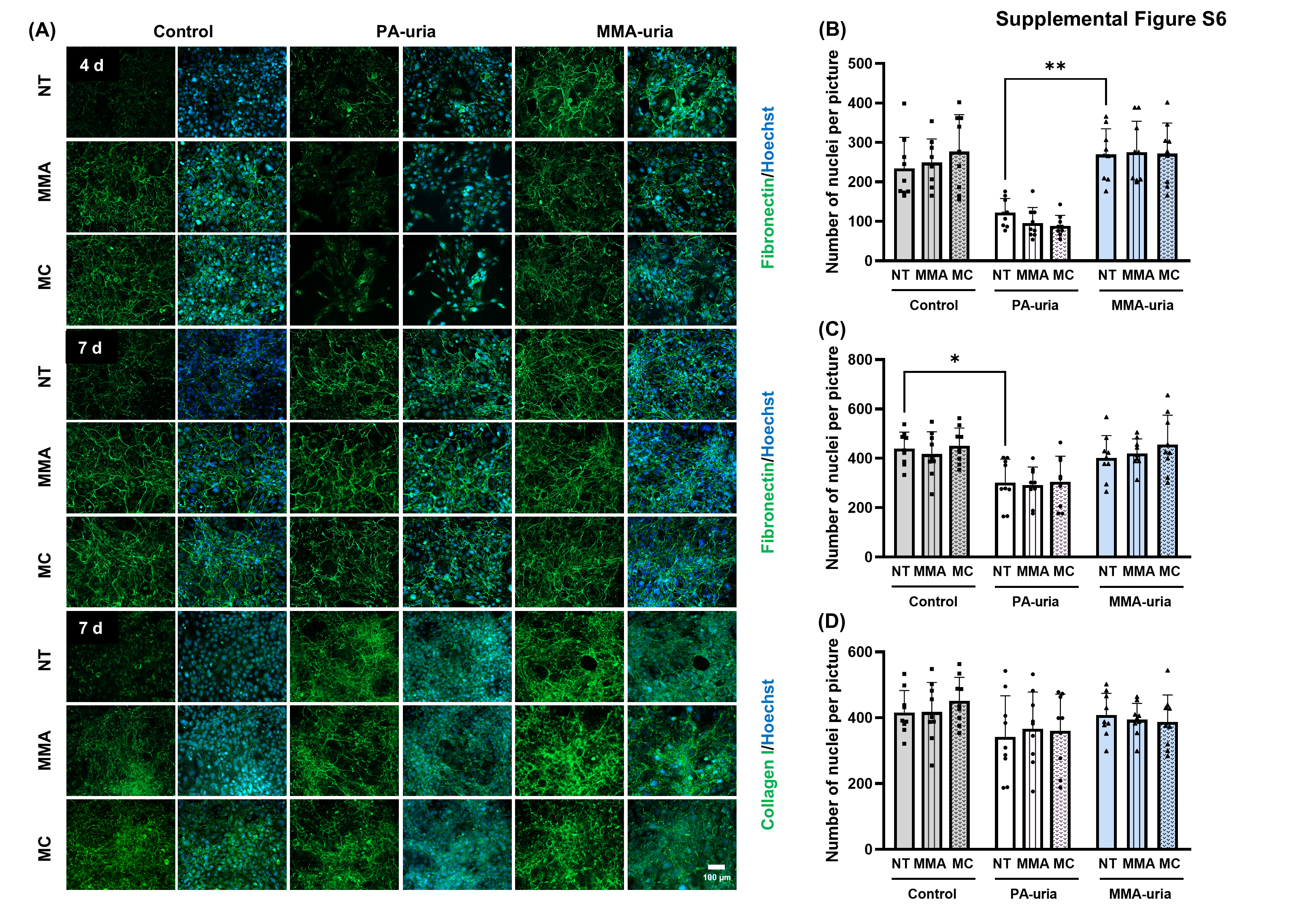

Supplement: Supplementary file 6 — Figure S6: (A) The immunofluorescent images from Figure 4 are shown along with merged images of Hoechst staining to account for phenotypic changes through different cell numbers. (B–D) Quantification of the number of nuclei per picture of the immunofluorescent staining in Figure 4, as shown by Hoechst staining after 4 days (B) and 7 days (C) for the fibronectin staining and after 7 days (D) for the collagen I staining. Data points represent three technical replicates for each of three biological replicates. [file JIMD-48-0-s008.png]

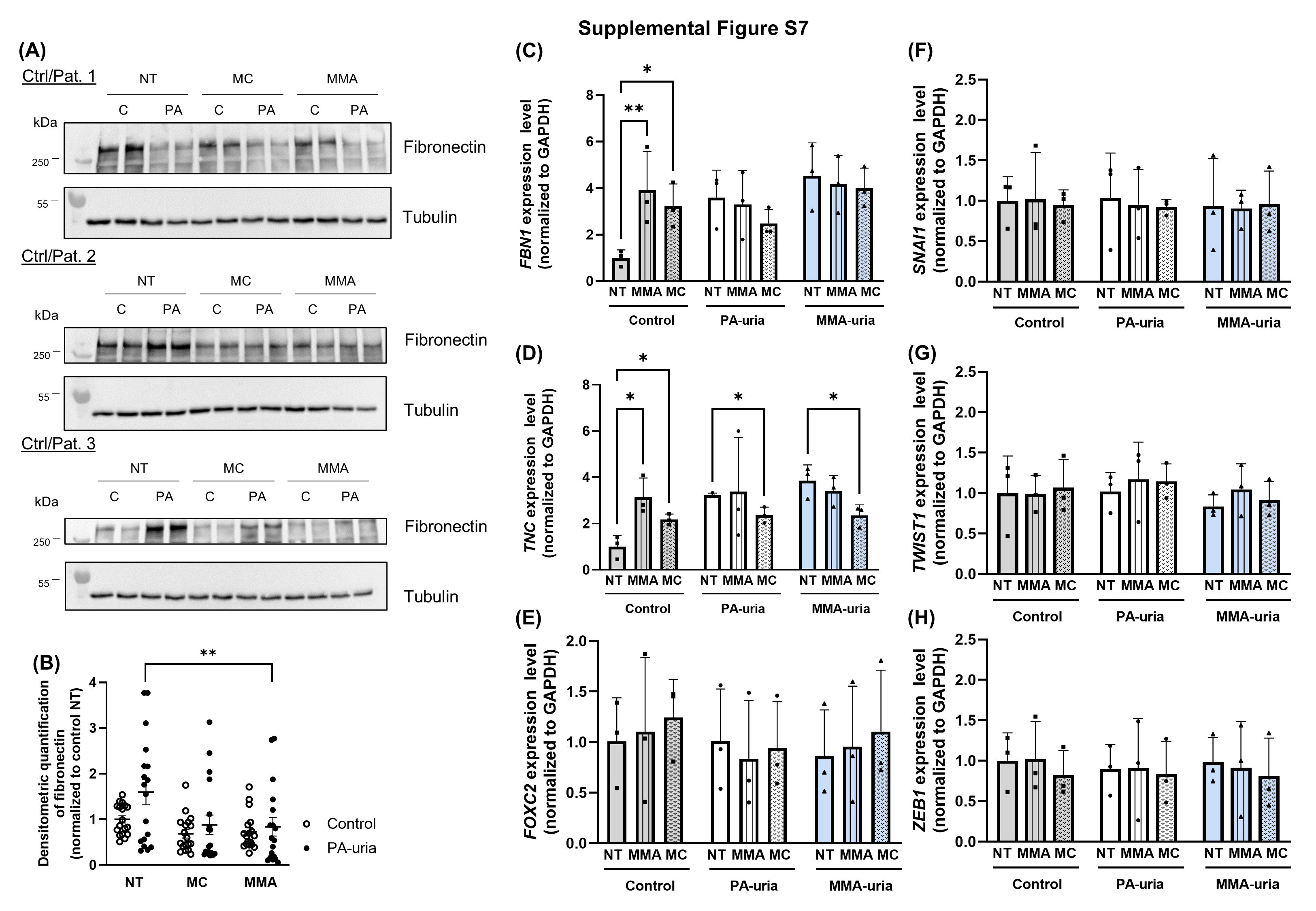

Supplement: Supplementary file 7 — Figure S7: (A) Western blotting of fibronectin in cell lysates from PA‐uria and control cells, either untreated (NT) or exposed to methylmalonic acid (MMA) or methylcitric acid (MC). Due to differences between patient cells, blots for each patient are shown. (B) Densitometric quantification of the Western blot in (A) relative to untreated control cells. n = 3. Data points represent three technical replicates for each of three biological replicates in two independent experiments. (C–H) Transcript levels of fibrillin‐1 (FBN1, C), tenascin C (TNC, D), forkhead box C2 (FOXC2, E), snail (SNAI1, F), and twist 1 (TWIST1, G) and T zinc‐finger‐enhanced binding protein 1 (ZEB1, H) were assessed by real‐time quantitative PCR renal epithelial cells from patients with PA‐uria or MMA‐uria and controls. Cells were either cultured under regular conditions (NT) or treated with MMA or MC. n = 3 per group, data points represent biological replicates. [file JIMD-48-0-s003.png]

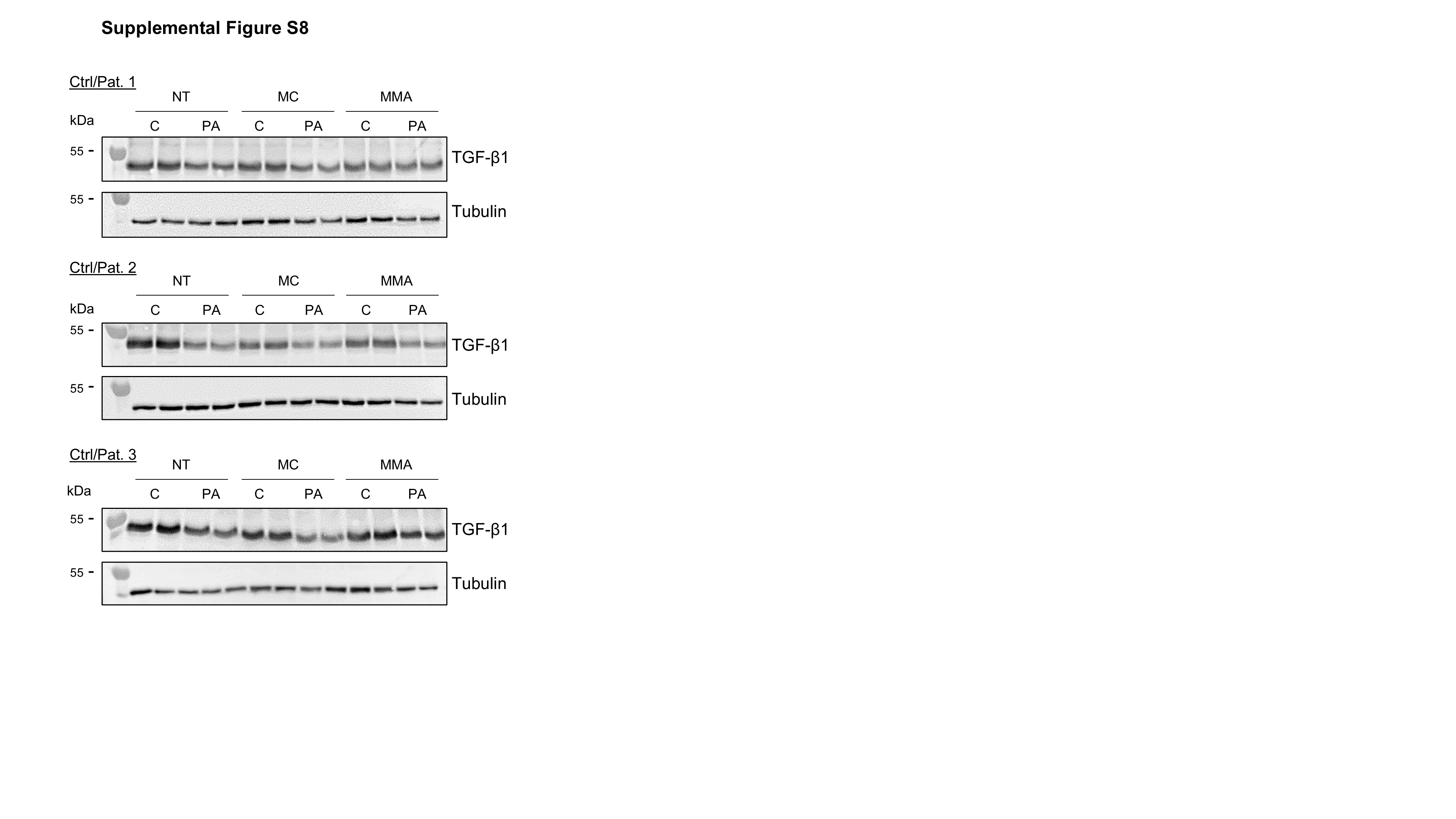

Supplement: Supplementary file 8 — Figure S8: Western blotting of TGF‐β1 in cell lysates from PA‐uria and control cells, either exposed to methylmalonic acid (MMA) or methylcitric acid (MC) or cultured under treatment conditions (NT). Blots from all three different patient PA‐uria cell lines and controls used are shown. [file JIMD-48-0-s007.png]

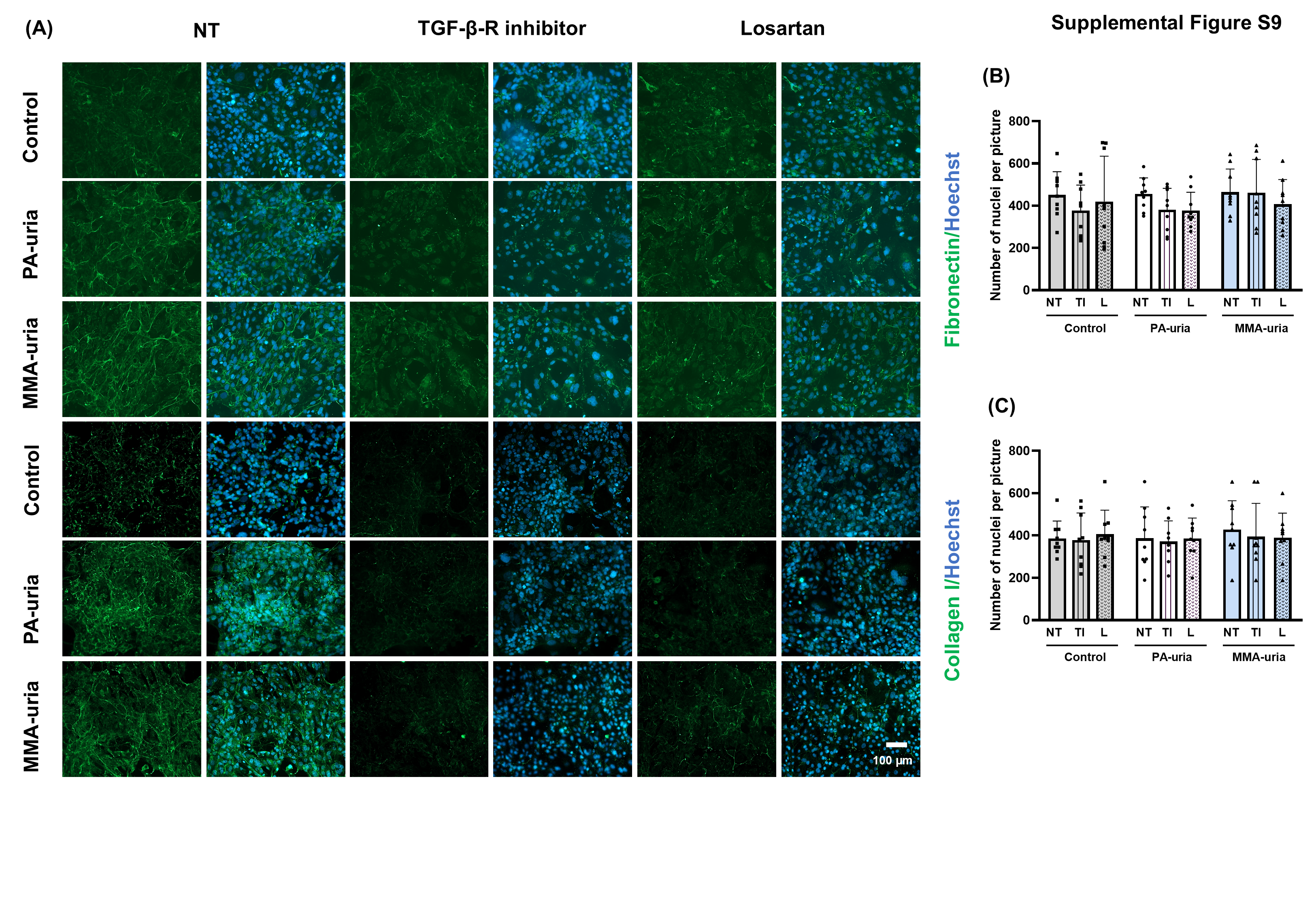

Supplement: Supplementary file 9 — Figure S9: (A) The immunofluorescent images from Figure 5 are shown along with merged images of Hoechst staining to account for phenotypic changes through different cell numbers. (B and C) Quantification of the number of nuclei per picture of the immunofluorescent staining in Figure 4, as shown by Hoechst staining after 7 days for both the fibronectin (B) and collagen I (C) staining. Data points represent three technical replicates for each of three biological replicates. [file JIMD-48-0-s006.png]

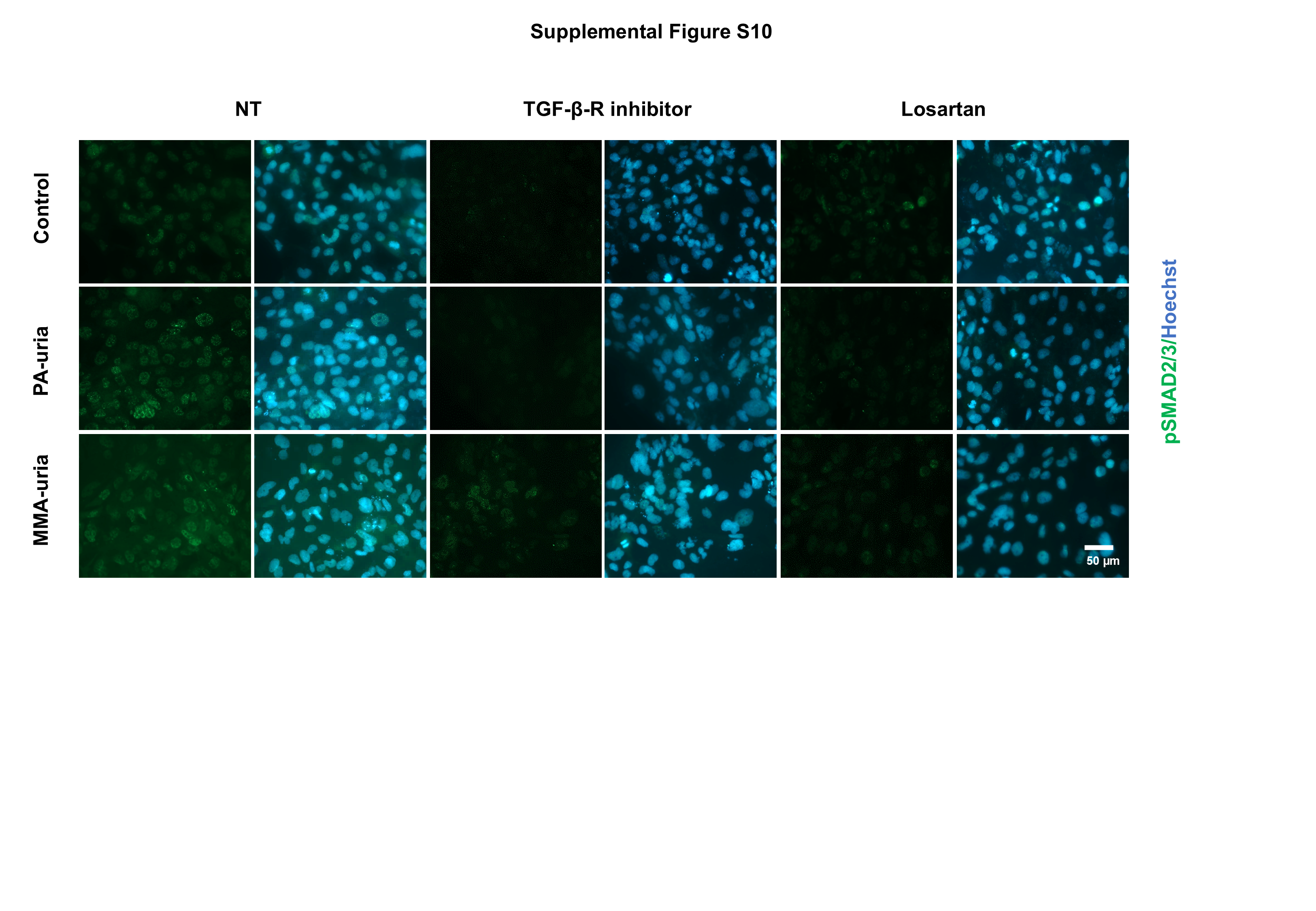

Supplement: Supplementary file 10 — Figure S10: Representative immunofluorescent staining for pSMAD2/3 in renal epithelial cells from PA‐uria or MMA‐uria patients and controls after 7 days. Cells were either cultured under regular conditions (NT) or treated with losartan (L) or a TGF‐β receptor inhibitor (TI). Scale bar: 50 μm. n = 3. [file JIMD-48-0-s010.png]
