# Supplementary material for: Transforming Growth Factor‐β‐Mediated Fibrotic Remodeling Drives Chronic Kidney Disease in Methylmalonic Aciduria and Propionic Aciduria—Identification of a New Therapeutic Target
Source: J Inherit Metab Dis. 2025 Oct 25;48(6):e70111. doi: 10.1002/jimd.70111 (PMC12553402; doi:10.1002/jimd.70111)
Supplement: Supplementary file 11 — Table S1: The disease‐causing variants on the gene and protein level are shown for the MMA‐uria and PA‐uria cell lines used. [file JIMD-48-0-s009.docx]

| **Cell line** | **Gene/ Class** | **Nucleotide change** | **Amino acid change** |
| --- | --- | --- | --- |
| *Control_1* | *-* | - | - |
| *Control_2* | - | - | - |
| *Control_3* | - | - | - |
| *MMA_1* | *MMUT* mut^0^ | c.607C>A; c.1105C>T | p.S288P; p.H386R |
| *MMA_2* | *MMUT* mut^0^ | c.862T>C; c.862T>C | p.S288P; p.S288P |
| *MMA_3* | *MMUT* mut^0^ | c.982C>T; c.982C>T | p.L328F; p.L328F |
| *PA_1* | *unknown* | unknown | unknown |
| *PA_2* | *PCCB* | c.1534C>T (homozygous) | p.R512C |
| *PA_3* | *PCCB* | c.76dupC; c.1127G>T | p.R26fs; p.R376L |

**Supplemental Table 1**
