# Supplementary material for: Transforming Growth Factor‐β‐Mediated Fibrotic Remodeling Drives Chronic Kidney Disease in Methylmalonic Aciduria and Propionic Aciduria—Identification of a New Therapeutic Target
Source: J Inherit Metab Dis. 2025 Oct 25;48(6):e70111. doi: 10.1002/jimd.70111 (PMC12553402; doi:10.1002/jimd.70111)
Supplement: Supplementary file 13 — Table S3: Sequences of forward and reverse primers used for real‐time quantitative PCR are listed for each gene. [file JIMD-48-0-s004.docx]

| **Gene** | **Forward primer (5’ – 3’)** | **Reverse primer (5’ – 3’)** |
| --- | --- | --- |
| *GAPDH* | CCCATCACCATCTTCCAG | ATGACCTTGCCCACAGCC |
| *LCN2* | CCACCTCAGACCTGATCCCA | CCCCTGGAATTGGTTGTCCTG |
| *HAVCR1* | TGTCTGGACCAATGGAACCC | GGCAACAATATACGCCACTGT |
| *FGF21* | GCCTTGAAGCCGGGAGTTATT | GTGGAGCGATCCATACAGGG |
| *AQP1* | CTGGGCATCGAGATCATCGG | ATCCCACAGCCAGTGTAGTCA |
| *AQP2* | GCTCCGCTCCATAGCCTTC | GGGTGCCAATACCCAAGCC |
| *FN1* | CGGTGGCTGTCAGTCAAAG | AAACCTCGGCTTCCTCCATAA |
| *COL1A1* | AGGGACACAGAGGTTTCAGTGGTT | GCAGCACCAGTAGCACCATCATTT |
| *FBN1* | TTTAGCGTCCTACACGAGCC | CCATCCAGGGCAACAGTAAGC |
| *TNC* | TCCCAGTGTTCGGTGGATCT | TTGATGCGATGTGTGAAGACA |
| *FOXC2* | CCTCCTGGTATCTCAACCACA | GAGGGTCGAGTTCTCAATCCC |
| *SNAI1* | TCGGAAGCCTAACTACAGCGA | AGATGAGCATTGGCAGCGAG |
| *TWIST1* | GAGCAAGATTCAGACCCTCAAG | CCATCCTCCAGACCGAGAAG |
| *ZEB* | CAGCTTGATACCTGTGAATGGG | TATCTGTGGTCGTGTGGGACT |
| *VIM* | AGTCCACTGAGTACCGGAGAC | CATTTCACGCATCTGGCGTTC |
| *CDH1* | AGTGACTGATGCTGATGCCC | GAACAGCTGTGAGGATGCCA |
| *TGFB1* | CAATTCCTGGCGATACCTCAG | GCACAACTCCGGTGACATCAA |
| *TGFB2* | CAGCACACTCGATATGGACCA | CCTCGGGCTCAGGATAGTCT |

**Supplemental Table 3**
